# Garlic oil-loaded nanodisks for the amelioration of acute lung injury via modulation of the NF-κB and Keap1–Nrf2 axis

**DOI:** 10.3389/fmolb.2025.1686436

**Published:** 2026-01-05

**Authors:** Ruilin Hou, Bowen Jiang, Kai Wang, Xiaoying Yang, Wenping Zhang

**Affiliations:** 1 Drug/Medical Device Clinical Trial Institution Office, General Hospital of Ningxia Medical University, Yinchuan, Ningxia, China; 2 Outpatient Department, General Hospital of Ningxia Medical University, Yinchuan, Ningxia, China; 3 Department of Pharmacy, Institute of Clinical Pharmacology, General Hospital of Ningxia Medical University, Yinchuan, Ningxia, China

**Keywords:** acute lung injury, garlic oil, inflammation, lipopolysaccharide, nanodisks, oxidative stress

## Abstract

**Background:**

Acute lung injury (ALI) and acute respiratory distress syndrome (ARDS) are prevalent and severe respiratory conditions with high morbidity and mortality rates, and specific treatment modalities are lacking. Garlic oil (GO), which is rich in sulfur compounds, has diverse biological properties, including anti-inflammatory and antioxidant effects; nonetheless, its utility is hindered by its limited water solubility and bioavailability. Nanotechnology-based formulations offer a promising solution to enhance GO efficacy. The aim of this investigation was to elucidate the protective effect and underlying mechanism of GO nanodisks (GO-nanodisks) on lipopolysaccharide (LPS)-induced acute lung injury.

**Methods:**

We developed a novel prescription utilizing GO-nanodisks. An acute lung injury model was induced in mice through LPS administration. The mice were randomly allocated into groups: healthy untreated, positive control, GO (50 mg/kg), and GO-nanodisks (50 mg/kg). Tail vein injections were administered accordingly. Subsequent assessments included lung histopathology; inflammatory cytokine (TNF-α, IL-6, IL-4, and IL-10) levels; oxidative stress marker (MDA, SOD, T-AOC, NO, and CAT) levels; and protein expression analyses.

**Results:**

This study successfully developed GO-nanodisks using a novel fabrication method. The GO-nanodisks demonstrated favorable physicochemical characteristics, with a mean particle diameter of 148 ± 3 nm, a polydispersity index (PDI) of 0.15 ± 0.02, a zeta potential of −0.2 ± 0.1 mV, and an encapsulation efficiency of 55.26% ± 0.04%. Compared with the positive control group, the GO-nanodisk group presented significantly reduced lung tissue pathology, lower inflammatory factor levels, and an improved oxidative stress status. Furthermore, the GO-nanodisk group displayed Keap1/Nrf2 signaling pathway activation and NF-kappa B pathway inhibition, surpassing the efficacy of the GO group.

**Conclusion:**

The results of this study demonstrate that the nanodisks formulation developed in this work effectively enables stable encapsulation of GO, enhances its bioavailability, and improves its protective efficacy against LPS-induced ALI. Furthermore, this formulation provides a promising theoretical foundation for the encapsulation of oil-based pharmaceuticals.

## 1 Introduction

Acute lung injury (ALI) and its more severe variant, acute respiratory distress syndrome (ARDS), represent some of the most critical respiratory pathologies. If not effectively managed, these conditions can result in irreversible acute respiratory failure and multiple organ dysfunction syndrome (MODS), both of which are associated with exceedingly high mortality rates. Clinically, ALI and ARDS are characterized by acute progressive respiratory failure, dyspnoea, refractory hypoxemia, diffuse pulmonary infiltrates, and pulmonary oedema (Long et al., 2022). The pulmonary aetiologies of ALI/ARDS frequently include bacterial or viral pneumonia, aspiration lung injury, multiple blood transfusions, and drug overdose. Conversely, extrapulmonary contributors predominantly encompass systemic inflammatory response syndrome (SIRS), sepsis, severe traumatic shock, and disseminated intravascular coagulation. Despite over five decades of research, the incidence and mortality rates of ALI/ARDS among critically ill patients remain alarmingly elevated, rendering it one of the most prevalent and significant complications encountered in intensive care units (ICUs) (Zhang et al., 2024; Laffey and Kavanagh, 2017). Annually, ALI/ARDS affects approximately 3 million individuals worldwide, constituting 10% of ICU admissions. A study conducted across 18 ICUs in China revealed that 3.6% of 18,793 patients were diagnosed with ALI/ARDS (Huang et al., 2020). Among these patients, 9.7% presented with mild ALI/ARDS, 47.4% with moderate ALI/ARDS, and 42.9% with severe ALI/ARDS, with an associated mortality rate of 46.3% (Máca et al., 2017). The incidence of ALI/ARDS is positively associated with advancing age; individuals aged 65 years and older experience prolonged mechanical ventilation duration and extended intensive care unit (ICU) stays, along with increased morbidity and higher all-cause mortality across diverse etiologies of ALI. The ongoing COVID-19 pandemic further exacerbates these age-associated disparities in the clinical response to ARDS following comparable exposures (Southern et al., 2017; Schouten et al., 2022). The economic and psychological burdens imposed by ALI/ARDS on society and affected individuals are substantial. Consequently, improving patient survival rates and mitigating the mortality of this critical clinical condition is highly important.

Garlic oil (GO) and its organosulfur constituents have attracted significant scholarly interest for their potential roles in the prevention and treatment of pulmonary diseases because of their notable bioactive properties. The pharmacological attributes of GO suggest its therapeutic efficacy in various conditions, including chronic obstructive pulmonary disease (COPD), pulmonary fibrosis, and lung cancer (Dorrigiv et al., 2020). Research indicates that GO can inhibit the release of proinflammatory cytokines, such as TNF-α, IL-6, and IL-1β, which may mitigate airway inflammation and enhance pulmonary function (Dilxat et al., 2024). The pronounced antioxidant capabilities of GO enable the scavenging of free radicals, thereby safeguarding lung tissue from oxidative damage, a critical factor in the prevention and management of pulmonary disorders (Rouf et al., 2020). Moreover, GO has been shown to regulate the MUC5B gene, which modulates airway mucus secretion, thereby contributing to the establishment of a protective barrier in the respiratory tract and potentially reducing the incidence of respiratory diseases (Bae et al., 2012). Additionally, its robust antiviral and antibacterial properties inhibit the proliferation of various respiratory pathogens, effectively preventing respiratory infections (Long et al., 2020). In particular, during the COVID-19 pandemic, the immune-enhancing effects of GO and its capacity to suppress viral replication garnered considerable attention (Elsebai and Albalawi, 2022). Nevertheless, despite the promising implications of GO in the management of pulmonary diseases, further exploration of its specific mechanisms of action and clinical applications is warranted. Future research should prioritize high-quality clinical trials to substantiate the safety and efficacy of GO. GO is characterized by high volatility, a strong odor, poor water solubility, and low bioavailability (Sunanta et al., 2023). Consequently, there is an urgent need to develop innovative strategies capable of effectively stabilizing GO, enhancing its applicability, and overcoming its inherent limitations, particularly its high volatility.

Nanomicelles are self-assembled structures that form spontaneously from amphiphilic polymers in solvent environments and are driven by intermolecular hydrogen bonding, electrostatic interactions, and van der Waals forces (Torchilin, 2001). These structures exhibit a variety of morphologies, with spherical forms being the most prevalent, followed by rod-like, vesicular, lamellar, tubular, discoidal, prismatic, and other more intricate configurations (Torchilin, 2001). Discoidal nanoparticles exhibit the highest accumulation in various organs and the lowest accumulation in the liver due to their rotational flexibility in blood flow, which facilitates enhanced adhesion to cell membranes and allows for the evasion of phagocytosis by liver macrophages. Therefore, morphology has increasingly emerged as a pivotal consideration in the design of nanocarriers (Decuzzi et al., 2010). Recently, bicellar lipid systems have become popularized because their rich morphology can be applied in biochemistry, physical chemistry, and drug delivery technology (Amengual et al., 2023). PEG-stabilized nanodisks are increasingly attracting attention as a solubilization platform for poorly water-soluble compounds and as novel drug delivery systems due to their flexible structural components and distinctive morphologies (Tahmasbi Rad et al., 2019; Matsuo et al., 2018). PEG demonstrates solubility in both hydrophilic and hydrophobic solvents, enabling its ability to interact not only with hydrophilic molecules such as albumin but also with hydrophobic compounds, including indocyanine green (Lee and Larson, 2016). Due to the presence of hydrophobic segments within its structure, it has been hypothesized that PEG may partially intercalate into the liquid crystalline or fluid phases of lipid bilayer membranes under elevated temperature conditions (Magarkar et al., 2012). The use of PEGylated cationic bicelles as carriers of paclitaxel has been shown to enhance drug delivery and cytotoxicity in the human cell line PC3(Steffes et al., 2020). Nanodisk-based tenofovir drug delivery is used to penetrate the brain for HIV-1 infection treatment (Garcia et al., 2022). VAP-conjugated nanodisks adapt to the developed tumour vasculature of the lung cancer microenvironment, making them promising nanocarriers for NSCLC-targeting therapy (Song et al., 2022). Discoidal rHDL composed of DMPC and two copies of an apolipoprotein scaffold that circumscribes the perimeter of the discoidal bilayer can be applied as a device for therapeutic delivery (Fox et al., 2021). Moreover, PS antibody-coated nanodisks demonstrated enhanced targeting specificity in the lungs of mice and reduced hepatic uptake compared to nanospheres (Muro et al., 2008).

Considering the distinct characteristics of PEG-stabilized bilayer nanodisks and their lack of use for oils drug-targeted delivery, bilayer nanodisks, consisting of DSPC, cholesterol and DSPE-MPEG2000, were first employed for GO delivery to ameliorate LPS-induced acute lung injury and investigate anti-inflammatory pathways. In this study, GO-encapsulated bilayer nanodisks (GO-nanodisks) were prepared and characterized in terms of morphology, size, encapsulation efficiency and stability. In addition, the potential of GO-nanodisks to augment the protective effects of GO on LPS-induced ALI was explored, and the associated mechanism was elucidated. This preliminary study provides more promising evidence that PEG-stabilized bilayer nanodisks might act as potential carriers for hydrophobic liquid drugs.

## 2 Materials and methods

### 2.1 Chemical reagents

The following compounds were used in this study: GO (Yuanye Co. Ltd., Shanghai, China. CAS: 8008-99-9), 1,2-Dioctadecanoyl-sn-glycero-3-phophocholine (DSPC) (A.V.T. Co. Ltd., Shanghai, China. CAS: 816-94-4), N-(Carbonyl-methoxypolyethylene glycol 2000)-1,2-distearoyl-sn-glycero-3-phosphoethanolamine (DSPE-MPEG2000) (A.V.T. Co. Ltd., Shanghai, China. CAS: 147867-65-0), cholesterol (A.V.T. Co. Ltd., Shanghai, China. CAS: 57-88-5), chloroform (Chronchem Co. Ltd., Sichuan, China; CAS: 67-66-3), methanol (Thermo Fisher Scientific, United States, CAS: 67-56-1), formic acid (Macklin, Shanghai, China, CAS: 64-18-6), 4-(2-hydroxyethyl)piperazine-1-ethanesulfonic acid (HEPES) (Amresco, CAS: 7365-45-9), potassium phosphate monobasic (Guangnuo Co. Ltd., Shanghai, China, CAS: 7778-77-0), potassium phosphate dibasic (Damao Co. Ltd., Tianjin, China, CAS: 7758-11-4), and dimethyl sulfoxide (DMSO) (Biotopped, Beijing, China, CAS: 67-68-5).

### 2.2 Preparation and characterization of garlic oil-loaded PEG-stabilized bilayer nanodisks

#### 2.2.1 Preparation of garlic oil-loaded PEG-stabilized bilayer nanodisks

GO-nanodisks were prepared using the thin-film dispersion method with a molar ratio of DSPC/cholesterol/MPEG_2K_-DSPE of 6:2:2 mol. Briefly, 15 mg of DSPC, 17.7 mg of DSPE-MPEG2000, 2.5 mg of cholesterol, 10 μL of GO and chloroform were placed in a flask. During gentle stirring for 2 h, the organic solvent was subsequently slowly evaporated using a rotary evaporator under reduced pressure at 40 °C. As the solvent volatilized, phospholipid molecules formed a thin and dense GO-loaded lipid film on the flask surface. The product was sealed to keep it dry and was stored at 4 °C for more than 3 days. Then 10 mL of ddwater was added to GO-loaded lipid film to facilitate dissolution, followed by sonication for 4 min/450 W utilizing an ultrasonic cell disruptor to prepare the GO-nanodisk formulation. Blank nanodisks were prepared using the same method as described above, but without incorporation of GO.

#### 2.2.2 Characterization of GO-loaded nanodisks

The solution was dissolved in selected solvents and processed by low-temperature ultrasonication to obtain nanodisks. The mean particle size, DPI and zeta potential of the nanodisks was analyzed using a Malvern Zetasizer Nano ZS90 (Malvern Nano ZS, Malvern, UK). Briefly, 1 mL of the GO nanomicelle mixture was placed in a dish and then placed on a Marvin laser particle size tester to determine the particle size distribution.

The morphology of blank disks and GO-nanodisks was examined using transmissionelectron microscopy (H-7650, Hitachi, Japan). A drop of nanoparticle suspension was visualized after staining with 2% (w/v) phosphotungstic acid for 30 s on a copper grid under TEM. Micrographs were acquired at a magnification of 20,000× with an accelerating voltage of 200 kV.

GO-loaded nanodisks were dissolved in deionized water, HEPES solution, or phosphate buffer (pH 5.8), and stability assessments were conducted at controlled temperatures (4 °C, room temperature, and 40 °C) to validate the encapsulation efficiency and stability. The GO concentration was quantitatively determined using high-performance liquid chromatography (HPLC, Agilent 1100 Series) following 1:1 mixing with methanol. After identifying the optimal solvent system, the micelles were reconstituted in deionized water and subjected to centrifugal separation at 14,000 rpm for 10 min, yielding a precipitated phase containing encapsulated GO. The precipitate was subsequently resolubilized in DMSO through sequential processing involving 5 min of vortex mixing and 30 min of sonication, with this dissolution cycle repeated three times to ensure complete micellar dissociation. Both the supernatant and the DMSO-dissolved precipitate fractions were mixed with the same volume of methanol prior to the HPLC analysis for a precise determination of the encapsulation efficiency (EE). The EE is calculated using the following equation: EE% = (1 − Cf/Ct) * 100%, where Cf represents the concentration of free (unencapsulated) drug, and Ct denotes the total drug concentration present in the nanoparticle or liposome suspension.

#### 2.2.3 Chromatographic condition

Chromatographic separation was carried out using isocratic elution on an C_18_ column (250 mm × 4.6 mm, 5 μm) within 30 min. Mobile phase was composed of methanol-water-formic acid (v/v, 85:15:0.1), at a flow rate of 0.8 mL/min and a UV detection wavelength of 228 nm (Li et al., 2006). The method exhibited linearity over the concentration range of 10–500 μg/mL. Linear regression analysis yielded the equation y = 36.0504151x +583.57966, with an r value of 0.99610.

### 2.3 Animal

Male ICR mice (18–22 g) were purchased from Beijing HFK Bioscience Co., Ltd., China. All animal experiments complied with National Guidelines for the Care and Use of Laboratory Animals (Laboratory Animal Guidelines for Ethical Review of Animal Welfare) (GB/T35892- 2018, China) and approved by the General Hospital of Ningxia Medical University Animal Care and Use Committee (Certificate number no: KYLL-2025-0079). The mice were maintained in a controlled environment featuring an automated 12-h dark‒light cycle, with the temperature regulated at 22 °C ± 2 °C and a relative humidity of 50%–60%. They were provided unrestricted access to a standard dry diet and tap water and were allowed to acclimatize for a minimum of 1 week prior to experimentation.

### 2.4 LPS-induced ALI model

The ALI model was established through a single intraperitoneal injection of LPS (O111:B4, Sigma‒Aldrich, United States) at a dosage of 15 mg/kg, as described previously (Su et al., 2020). 6 h after injection, the mice received an intravenous injection of either 50 mg/kg GO-nanodisks (available on demand) or GO (dissolve in a mixture of 5% absolute ethanol and 95% NS). The mice were randomly allocated into the following experimental groups (n = 10): healthy untreated, positive control, LPS + GO-nanodisks (50 mg/kg, 8 h after LPS), and LPS + GO (50 mg/kg, 8 h after LPS). 2 h following the intravenous administration of GO, the animals were anesthetized via intraperitoneal injection of pentobarbital at a dose of 60 mg/kg. Subsequently, terminal anesthesia was induced using an intraperitoneal injection of pentobarbital at 150 mg/kg, ensuring compliance with endpoints for minimizing distress. Efforts were made to minimize animal suffering and discomfort, as well as to reduce the number of animals used to the extent possible (Percie du Sert et al., 2020). Thereafter, lung tissue samples, blood, and bronchoalveolar lavage fluid (BALF) were collected, in accordance with established endpoints designed to minimize animal distress.

### 2.5 Lung wet/dry weight ratio and lung coefficient

The body and wet whole-lung tissue weights of the mice were recorded, and the lung coefficient was calculated. The upper lobe of the left lung was excised and washed with PBS, after which its wet weight was immediately measured. The lung tissues were subsequently dried at 65 °C for 72 h, and the weight of the dried lung was recorded. The lung coefficients (wet whole-lung tissue weights/body weight *100%) and the wet-to-dry (wet weight/dry weight, W/D) weight ratio of the lungs were assessed to evaluate the severity of pulmonary oedema in the mice.

### 2.6 Histological evaluation

The left lung tissues were washed with prechilled PBS, fixed with formalin, embedded in paraffin, and sliced into 5 µm sections. The samples were stained with haematoxylin and eosin (H&E) and examined microscopically (Olympus Corporation, Tokyo, Japan). The pathological scores for lung injury were assessed according to previously established criteria (Su et al., 2020). The histological characteristics of the lung injury (including alveolar edema and hemorrhage, the number of infiltrating leukocytes, and the thickness of the alveolar wall and epithelium) were evaluated. Each histological characteristic was evaluated on a scale of 0–3 (0, normal; 1, mild; 2, moderate; 3, severe).

### 2.7 BALF collection and protein concentration analysis

Following euthanasia, the chest cavity was accessed, and the left bronchus was ligated. A sterile, blunt-ended needle was used to inject 0.6 mL of cold PBS into the right lung lobe for the purpose of collecting BALF, repeated three times. Each mouse yields approximately 1.5 mL of BALF, collected in a single 1.5 mL centrifuge tube. The total protein concentration in the BALF was determined using a bicinchoninic acid (BCA) protein assay kit (SW201-02, Seven Biotech, Beijing, China) in accordance with the manufacturer’s instructions to evaluate lung permeability.

### 2.8 Enzyme-linked immunosorbent assay (ELISA) analysis

Lung tissue lysates were prepared using ice-cold PBS at 4 °C, followed by centrifugation at 12,000 rpm for 10 min. In accordance with the manufacturer’s instructions, the concentrations of tumour necrosis factor-alpha (TNF-α), interleukin-4 (IL-4), interleukin-6 (IL-6), and interleukin-10 (IL-10) in BALF and lung tissues were subsequently quantified with mouse ELISA kits (catalogue numbers 88-7324, 88-7044, 88-7064, and 88-7105, Thermo Fisher Scientific, United States).

### 2.9 Measurement of nitric oxide (NO) release

A nitric oxide assay kit (S0021, Beyotime, Shanghai, China) was used to evaluate the levels of NO *in vivo*. The concentration of NO was determined by measuring the levels of nitrates and nitrites using the Griess method. All samples were prepared in accordance with the manufacturer’s guidelines.

### 2.10 Detection of oxidative stress in ALI model

Lung tissues were initially immersed in ice-cold PBS at 4 °C, followed by rapid homogenization and centrifugation at 12,000 rpm for 10 min to isolate the supernatant for subsequent analysis. The total antioxidant capacity (T-AOC) was assessed using a Total Antioxidant Capacity Assay Kit employing the rapid ABTS method (S0121, Beyotime, Shanghai, China). The levels of superoxide dismutase (SOD) were quantified using the Total Superoxide Dismutase Assay Kit with WST-8 (S0101S; Beyotime, Shanghai, China). Additionally, the concentration of malondialdehyde (MDA) was measured using the Lipid Peroxidation MDA Assay Kit (S0131S, Beyotime, Shanghai, China), whereas hydrogen peroxide (H_2_O_2_) levels were determined using the Catalase Assay Kit (S0051, Beyotime, Shanghai, China). All analyses were conducted in accordance with the manufacturer’s protocols.

### 2.11 Western blot analysis and antibodies

Total proteins from the lung tissues were extracted using ice-cold lysis buffer (KGP2100, KeyGEN BioTECH, Nanjing, China). Cytoplasmic and nuclear protein extracts were prepared from fresh tissue samples following the manufacturer’s instructions (sc-003, Invent Biotechnologies, Inc., United States). The samples were centrifuged at 14,000 rpm for 5 min at 4 °C. Following centrifugation, the protein supernatants were collected, and protein concentrations were quantified using the BCA assay, with the samples subsequently stored at −80 °C. A total of 40 μg of protein and 35 μg of nuclear protein were separated using sodium dodecyl sulfate‒polyacrylamide gel electrophoresis (SDS–PAGE) on 10% gels (SW143-02, Seven Biotech, Beijing, China) and subsequently transferred to a polyvinylidene fluoride (PVDF) membrane (A30705986, Cytiva, Shanghai, China). The membranes were then blocked with 5% nonfat dry milk (SW128‒03, Seven Biotech, Beijing, China) for 2 h at room temperature. The blots were subsequently incubated overnight at 4 °C with primary antibodies against p-p65 (AF 2006, Affinity Biosciences, United States), p65 (10745-1-AP, Proteintech, Wuhan, China), Nrf2 (16396-1-AP, Proteintech, Wuhan, China), NQO1 (11451-1-AP, Proteintech, Wuhan, China), HO-1/HMOX1 (10701-1-AP, Proteintech, Wuhan, China), Lamin B1 (12987-1-1AP, Proteintech, Wuhan, China), and GAPDH (10494-1-AP, Proteintech, Wuhan, China). After three washes with PBST, the samples were incubated with a secondary antibody (SA00001-2; Proteintech, Wuhan, China) for 1 h. Finally, the signals were detected using enhanced chemiluminescence (ECL) reagents (SW134-01; Seven Biotech, Beijing, China).

### 2.12 Statistical analysis

Statistical analyses included one - way ANOVA. All the data are presented as means ± SEMs, and GraphPad Prism version 10.0 software (GraphPad Software, Inc., San Diego, CA, United States) was used for data analysis. After the ANOVA analysis, the *post hoc* multiple comparisons were performed by using Tukey’s honestly significant difference test to determine the statistical difference from each other among subgroups unless otherwise indicated. *p* < 0.05 was considered significant, and n. s indicates not significant.

## 3 Result

### 3.1 Preparation and characterization of garlic oil -loaded PEG-stabilized bilayer nanodisks

**FIGURE 1:**
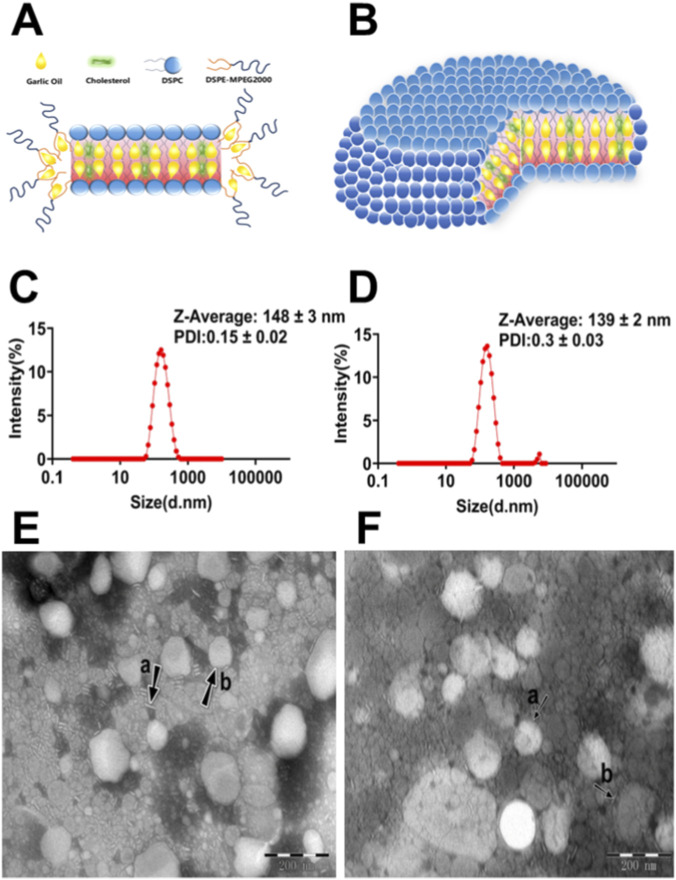
Preparation and characterization of garlic oil -loaded PEG-stabilized bilayer nanodisks. Planar **(A)** and spatial **(B)** structures of GO-nanodisks. Lipids with light blue head groups symbolize phospholipids, those with dark blue head groups represent PEG-lipids, the yellow parts represent GO, and the green parts symbolize cholesterol. Dynamic light scattering analysis of GO-nanodisks **(C)** and blank-nanodisks **(D)**. TEM images of the GO-nanodisks **(E)** and blank-nanodisks **(F)**. The black arrows and arrowheads denote disks observed face-on and edge-on, respectively. The scale bar on the TEM image is 200 nm and 20 k magnification.

GO-nanodisks were prepared by thin-film dispersion in combination with the sonication hydration method. GO was incorporated into the hydrophobic region of the disks. The nanostructure of the GO-nanodisks is schematically shown in Figures 1A,B. The disks are open bilayer structures, with a hydrophobic discoid centre built by DSPC/cholesterol and a hydrophilic PEG edge covering the hydrophobic rim by DSPE-MPEG2000. As shown in the diameter distribution diagram and TEM image in Figures 1C,E, the GO-nanodisks were successfully prepared, with essentially disk-shaped morphologies. The mean particle diameter was 148 ± 3 nm, the polydispersity index (PDI) was 0.15 ± 0.02 and the zeta potential of the GO-nanodisks was −0.2 ± 0.1 mV based on three independent replicates. The encapsulation efficiency was as high as 55.26% ± 0.04%. Due to the higher density of GO than that of the ddwater, the stability of the preparation was improved, and the concentration of the GO-nanodisks was increased through centrifugation. Several studies have integrated dynamic light scattering (DLS) with TEM to conduct comparative analyses aimed at characterizing the particle size and morphology of nanoformulations in Supplementary Figure S1 (Filippov et al., 2023). Figures 1D,F present the size distribution and morphological characteristics of blank (unloaded) nanodisks, respectively. Analysis of data obtained from three independent replicate experiments revealed that the average particle diameter was 139 ± 2 nm, PDI was 0.3 ± 0.03, and the zeta potential of the GO-nanodisks was −0.2 ± 0.03 mV.

### 3.2 GO-nanodisks effectively ameliorated LPS-induced ALI

**FIGURE 2:**
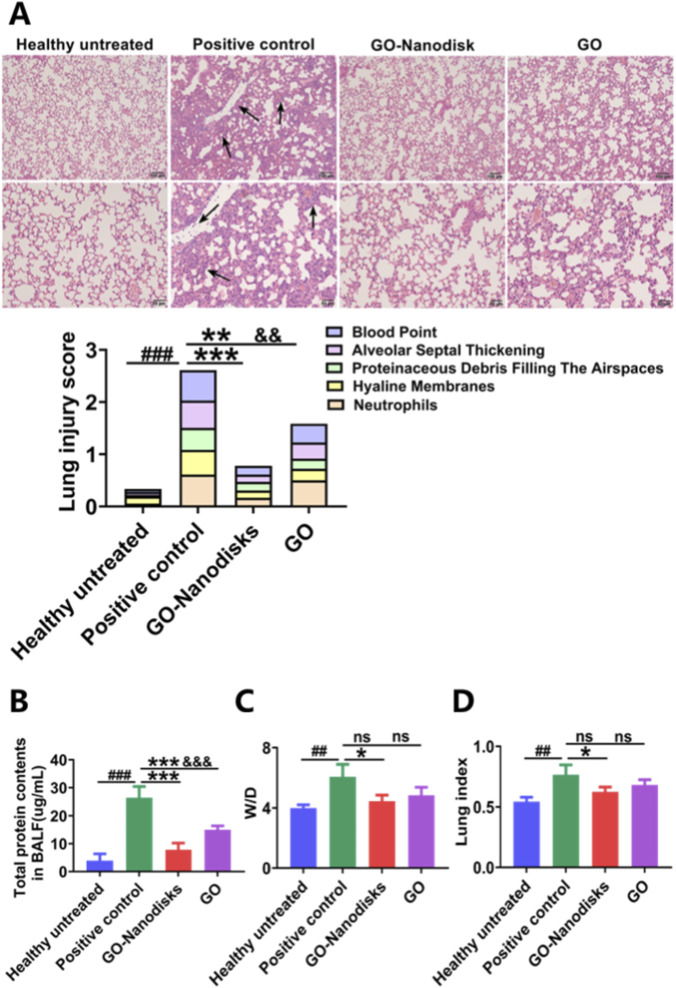
Protective effects of GO-nanodisks against LPS-induced ALI in mice. **(A)** Representative H&E-stained lung tissue sections (magnification 10x, 20x and scale bar 100 μm, 50 μm). The black arrows denote inflammatory cell infiltration, congestion and oedema, were observed in the thickened alveoli. **(B)** Total protein concentration in BALF. **(C)** Lung wet-to-dry (W/D) weight ratio. **(D)** Lung index. Data are expressed as mean ± SD (n = 6). *##p* < 0.01, *###p* < 0.001 vs. healthy untreated group; *p < 0.05, **p < 0.001 vs. positive control group; &&&*p* < 0.001 vs. GO-nanodisks; ns, GO group vs. positive control group or GO-nanodisks group, using one-way ANOVA.

The alveolar architecture in the healthy untreated group remains intact, exhibiting well-defined boundaries and showing no evidence of pathological abnormalities. The positive control mice exhibited obvious pathological features of ALI. Many inflammatory cells accumulated in the pulmonary interstitium; the alveolar septum thickened and the integrity of the alveoli was damaged. Obvious intra-alveolar haemorrhage and congestion, such as inflammatory cell infiltration, congestion and oedema, were observed in the thickened alveoli, as shown in Figure 2A black arrows. In contrast, the alveolar structures of the GO-nanodisk and GO groups were relatively intact, with a significant reduction in inflammatory cell infiltration. Slight alveolar thickening and fewer bleeding points or congestion were observed. The mouse lung injury score was calculated, and the results revealed that the GO-nanodisks and GO had obvious protective effects on the lung tissue. And, the GO-nanodisks were more effective than the original GO. In addition, LPS significantly increased the protein content in the BALF of the mice, whereas treatment with 50 mg/kg GO-nanodisks significantly reduced the LPS-induced increase in the protein content in BALF (*p* < 0.001), as shown in Figure 2B. As shown in Figures 2C,D, the degree of pulmonary oedema was investigated by evaluating the W/D ratio (*p* < 0.05) and lung coefficient (*p* < 0.05). The results showed that the lung coefficient and W/D ratio in the positive control group increased significantly, indicating the occurrence of pulmonary congestion and pulmonary oedema. Compared with those in the positive group, the mouse lung coefficient and wet-to-dry weight ratio of the lungs in the GO-nanodisk (50 mg/kg) group were significantly decreased, indicating that the pulmonary congestion and pulmonary oedema of the mice were improved. Althougu no statistically significant differences were observed in the W/D and lung coefficient between the GO-nanodisk group and the GO group, the protective effect of GO-nanodisks was enhanced by approximately 1.1-fold relative to GO. The absence of statistical significance may be attributed to the limited sample size. These findings confirm that GO-nanodisks can reduce lung swelling and play a protective role in mice with ALI.

### 3.3 Evaluation of the antioxidant activity of GO-nanodisks in ALI mice

**FIGURE 3:**
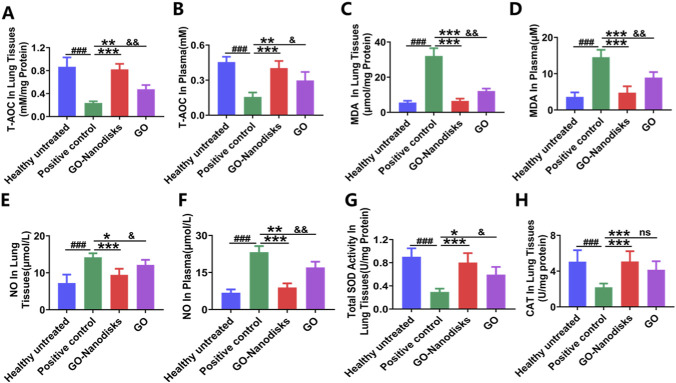
Evaluation of the antioxidant effects of GO-nanodisks in LPS-induced ALI in mice. Oxidative stress markers were measured in lung tissue and serum, including **(A,B)** total antioxidant capacity (T-AOC), **(C,D)** malondialdehyde (MDA), **(E,F)** nitric oxide (NO), **(G)** superoxide dismutase (SOD), and **(H)** catalase (CAT). Data are presented as mean ± SD (n = 6). ###*p* < 0.001 vs. healthy untreated group; *p < 0.05, **p < 0.01, **p < 0.001 vs. positive control group; &*p* < 0.05, &&*p* < 0.01 vs. GO group; ns, GO group vs. GO-nanodisks group, using one-way ANOVA.

Lung tissues and serum samples were collected for subsequent analysis to assess the therapeutic efficacy of the nanodisks in comparison with GO for the treatment of LPS-induced acute lung injury. The administration of GO-nanodisks at a dosage of 50 mg/kg mitigated the oxidative stress associated with LPS-induced ALI and increased the activity of antioxidant enzymes. The antioxidant properties of GO-nanodisks were evaluated based on its effects on oxidative stress induced by LPS. Notably, compared with those in the healthy untreated group, the reactive oxygen species (ROS) levels in both plasma and lung tissues from the positive control group were significantly increased (*p* < 0.001). Conversely, GO-nanodisks treatment resulted in a reduction in ROS levels, the restoration of the total antioxidant capacity (T-AOC) (Figures 3A,B, *p* < 0.001), and a decrease in malondialdehyde (MDA) levels (Figures 3C,D, *p* < 0.001) in the mice. These findings suggest that GO-nanodisks effectively mitigates oxidative stress by attenuating lipid peroxidation associated with LPS-induced ALI than GO (*p* < 0.05, *p* < 0.01). Furthermore, the activities of key antioxidant enzymes, specifically superoxide dismutase (SOD) and catalase (CAT), were significantly lower in the positive control group than in the GO-nanodisks group (Figures 3G,H, *p* < 0.001). The progression of oxidative stress was found to facilitate the production of nitric oxide (NO), prompting further investigations into the NO concentrations in murine subjects. The result was GO-nanodisks group could significantly reduce NO in ALI mice than GO group (Figures 3E,F, *p* < 0.05). Collectively, these results suggest that GO-nanodisks may alleviate oxidative stress induced by LPS by increasing the activities of selected antioxidant enzymes (SOD and CAT) in the lungs, thereby reducing the levels of MDA (a marker of oxidative stress) and NO. Encapsulation by GO-nanodisks markedly enhances the aqueous solubility of the parent drugs and prevents their precipitation in the bloodstream. Concurrently, the hydrophilic shell acts as a protective barrier, minimizing interactions between the parent drugs and blood-borne enzymes or proteins. This protective effect reduces the likelihood of drug degradation, thereby facilitating the delivery of a greater proportion of active drug molecules to pulmonary tissues via systemic circulation (Rahdar et al., 2021; Li et al., 2022). This approach can enhance the antioxidant capacity of the garlic oil technical material by approximately 60%.

### 3.4 GO-nanodisks attenuated LPS-induced multi-cytokine releases in mice

**FIGURE 4:**
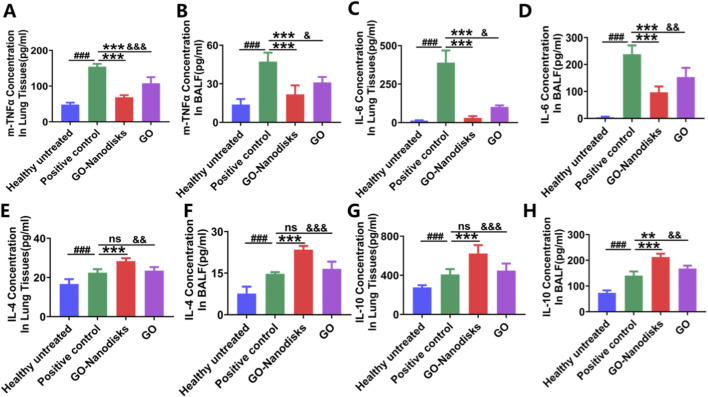
Evaluation of the effects of GO-nanodisks on LPS-induced cytokine release in mice with ALI. Cytokine levels were measured in bronchoalveolar lavage fluid (BALF) and lung tissue, including pro-inflammatory cytokines TNF-α **(A,B)** and IL-6 **(C,D)**, as well as anti-inflammatory cytokines IL-4 **(E,F)** and IL-10 **(G,H)**. Data are presented as mean ± SD (n = 6). ###*p* < 0.001 vs. healthy untreated group; **p < 0.01, **p < 0.001 vs. positive control group; &*p* < 0.05, &&*p* < 0.01, &&&*p* < 0.001 vs. GO group; ns, GO group vs. positive control group, using one-way ANOVA.

Inflammation is a pivotal factor in the pathophysiology of ALI/ARDS, as it can lead to both direct and indirect damage to the alveolar epithelium and pulmonary microvascular endothelium. This inflammatory response is a primary contributor to the development of ALI/ARDS (Zhu et al., 2013). Compared with inflammatory factor in the healthy untreated group, the levels of proinflammatory cytokines, specifically TNF-α and IL-6, in lung tissues and BALF were markedly increased in the positive control group (Figures 4A–D, *p* < 0.001). Additionally, LPS stimulation led to a modest increase in the levels of IL-4 and IL-10 in both BALF and lung tissues company (Figures 4E–H), compared with healthy untreated group (*p* < 0.001). However, treatment with 50 mg/kg GO-nanodisks resulted in a significant reduction in the levels of these inflammatory cytokines (*p* < 0.001). Meanwhile, GO-nanodisks significantly increased the levels of anti-inflammatory cytokines (*p* < 0.001). Notably, the efficacy of GO was markedly improved when GO was administered in nanodisks form at the same concentration and using the same method, with a statistically significant difference observed (*p* < 0.05). In the present study, the administration of GO-nanodisks demonstrated a substantial suppressive effect on the cytokine storm in a murine model and was able to augment the anti-inflammatory efficacy of the parent compound, GO, by approximately 40%.

### 3.5 GO-nanodisks treatment decreases oxidative stress by activating Keap1-Nrf2 pathways in ALI

**FIGURE 5:**
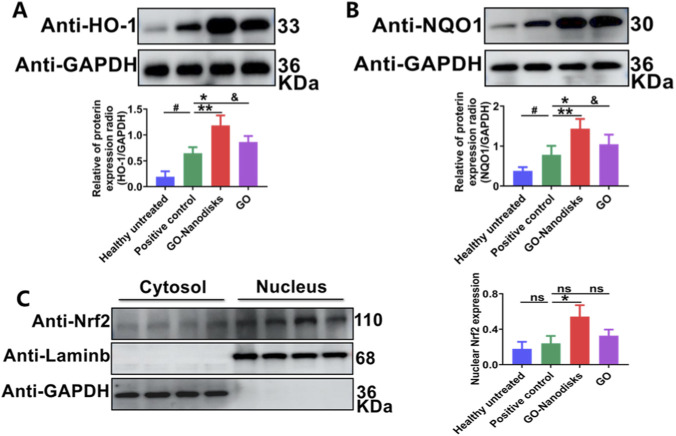
Effect of GO-nanodisks on the activation of the Keap1–Nrf2 signaling pathway in ALI mice. Protein expression levels of HO-1 **(A)**, NQO1 **(B)**, and nuclear Nrf2 (nu-Nrf2), **(C)** in lung tissues were assessed by Western blot analysis. Data are presented as mean ± SD (n = 3). Statistical significance: #*p* < 0.05 vs. healthy untreated group; *p < 0.05, **p < vs. positive control group; &*p* < 0.05 vs. GO group; ns, GO group vs. positive control group or GO-nanodisks group, using one-way ANOVA.

The Keap1‒Nrf2 signaling pathway serves as the primary antioxidant defence mechanism against damage induced by environmental stressors. This pathway encompasses numerous upstream and downstream proteins, several of which were selected for investigation regarding the antioxidant properties of GO-nanodisks. The aim of this experiment was to assess the protein expression levels of HO-1 and NQO1, as well as the nuclear translocation of Nrf2, to determine the impact of GO-nanodisks on the Keap1‒Nrf2 signaling pathway. This approach was employed to determine whether the inhibitory effects of GO-nanodisks on LPS-induced oxidative stress are associated with the Keap1-Nrf2 signaling pathway. The experimental findings revealed that treatment with GO-nanodisks significantly increased the expression of the downstream proteins HO-1 and NQO1 in lung tissues (Figures 5A,B, *p* < 0.01). Furthermore, *in vivo* analyses revealed that the Nrf2 protein in the lung tissues of the GO-nanodisks group had significantly translocated from the cytoplasm to the nucleus (Figure 5C, *p* < 0.05). These results suggest that GO-nanodisks may mitigate LPS-induced oxidative stress by enhancing the activation of the Keap1-Nrf2 signaling pathway. Oxidative damage to pulmonary tissue predominantly transpires within intracellular compartments, including alveolar epithelial cells and pulmonary macrophages (Bezerra et al., 2023). Although GO may be delivered to lung tissue, its lipophilic nature potentially impedes its ability to traverse the cellular membrane and gain intracellular access, thereby limiting its interaction with reactive ROS targets. In contrast, GO-nanodisks possess hydrophilic shells that facilitate cellular uptake, thereby enhancing the intracellular delivery of the therapeutic agent and enabling it to exert antioxidant effects directly within the cells.

### 3.6 GO-nanodisks alleviates LPS-induced ALI and inflammation through NF-κB pathways

**FIGURE 6:**
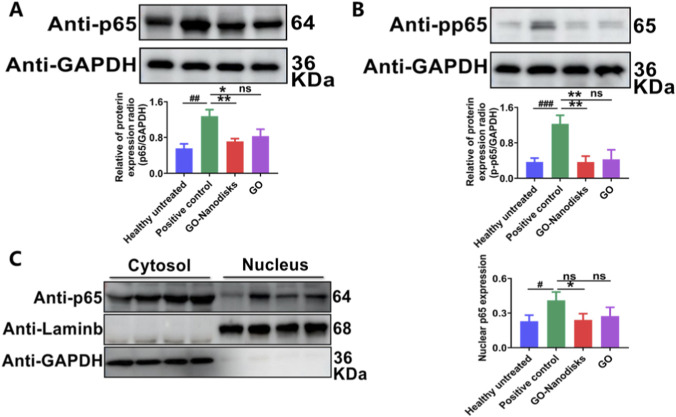
Effect of GO-nanodisks on the activation of the NF-κB pathway in ALI mice. Protein expression levels of p65 **(A)**, p-p65 **(B)** and nuclear p65 (nu-p65), **(C)** in lung tissues were assessed by Western blot analysis. Data are presented as mean ± SD (n = 3). Statistical significance: #*p* < 0.05. ##*p* <. ###*p* < 0.001 vs. healthy untreated group; #*p* < 0.05, ##*p* < 0.01 vs. positive control group; ns, GO group vs. positive control group or GO-nanodisks group, using one-way ANOVA.

Nuclear factor kappa-light-chain-enhancer of activated B cells (NF-κB) serves as a crucial transcriptional regulator in cells that participates in the inflammatory response. The activation of NF-κB leads to the expression of various genes and the subsequent production of cytokines, which can result in a cytokine storm. The aim of this experiment was to explore the relationship between the anti-inflammatory effects of GO-nanodisks and the NF-κB signaling pathway in the context of LPS-induced ALI. Western blot analysis revealed that LPS stimulation significantly increased the protein levels of p65 and phosphorylated p65 (p-p65) in lung tissues (*p* < 0.01). However, following the administration of GO-nanodisks the LPS-induced increases in the p65 and p-p65 protein levels were notably inhibited (Figures 6A,B, *p* < 0.01). Furthermore, GO-nanodisks had a protective effect and facilitated the repair of damaged lung tissues by significantly reducing the nuclear translocation of p65 in response to LPS stimulation (*p* < 0.05). These findings indicate that GO-nanodisks may mitigate the inflammatory response in LPS-induced ALI in mice by the NF-κB signaling pathway.

## 4 Discussion

The COVID-19 pandemic has prompted a re-evaluation of the definitions of ALI and ARDS. Autopsy findings from the first patient diagnosed with COVID-19 indicated that ARDS serves as the pathological foundation for severe manifestations of the disease. The case fatality rate among COVID-19 patients suffering from ARDS can exceed 70% (Xu et al., 2020). Currently, the primary therapeutic approach involves supportive care through mechanical ventilation, which is frequently employed to alleviate hypoxemia in patients with ALI/ARDS. However, the inappropriate application of mechanical ventilation may lead to complications such as the overexpansion or localized atelectasis of lung tissue, which can provoke and intensify the inflammatory response, potentially resulting in additional lung injury (Wick et al., 2024). Nevertheless, an increasing number of researchers contend that glucocorticoids do not significantly reduce mortality rates and may predispose patients to infections and other serious adverse effects. Consequently, the quest for safe and effective pharmacological agents for the prevention and treatment of ALI/ARDS remains a prominent focus in clinical research (Zhuang et al., 2020). GO, a derivative of garlic, contains various organic sulfur compounds, including diallyl sulfide, diallyl disulfide, and diallyl trisulfide, which exhibit a range of biological activities, such as antioxidant, antitumour, anti-inflammatory, renoprotective, hepatoprotective, and antiobesity effects (Bar et al., 2022; Hernández-Cruz et al., 2022). LPS, a component of the outer membrane of gram-negative bacteria, can induce direct lung injury when introduced into the airway and systemic effects when disseminated throughout the body, making it a reliable and stable model for inducing ALI in murine subjects (Menezes et al., 2005). In this study, a single intraperitoneal injection of LPS (15 mg/kg) was administered to establish the ALI model, effectively replicating acute damage to the pulmonary epithelial and endothelial barriers, as well as the acute inflammatory response within the airway in a short time frame (Zhang et al., 2018; Matute-Bello et al., 2008).

Bilayer disks, so-called bicelles, can be prepared from a mixture of medium- and short-chain phospholipids and other lipids (Johansson et al., 2005; van Dam et al., 2004), such as DHPC/DMPC and PEG-DSPE/DSPC/cholesterol. PEG lipids are mainly able to transform nanomicelles from a spherical to discoid shape because spherical vesicles present a window of elevated chain-melting temperatures and lipid packing shapes (Viitala et al., 2019). Here, a PEG-lipid concentration of 20 mol% was used to prepare GO-loaded nanodisks, as reported previously (Zhang et al., 2014). Because GO is an oil component, the encapsulation efficiency of GO-nanodisks is relatively low (36.97% ± 0.03%). The addition of cholesterol can enhance the encapsulation efficiency of GO-nanodisks to some extent. Cholesterol is well known to affect the membrane permeability and several studies have confirmed its ability to reduce the partitioning of drugs into phospholipid liposomes in a concentration dependent manner. The reduced cholesterol content in the brush border membrane vesicles appears to be compensated by other lipid components capable of acting either individually or in conjunction with cholesterol to reduce the propensity of drugs to partition into the membrane (Mardešić et al., 2023). However, achieving a satisfactory encapsulation efficiency remains a challenge when oil drugs are incorporated into disk-shaped micelles. In our subsequent studies, we aimed to improve the encapsulation efficiency of these micelles by adjusting the compositions and proportions of various lipids. Nanodisks exhibit prolonged circulation time, enhanced vascular margination, reduced phagocytosis rates, and greater tumor cell internalization in biological systems relative to spherical counterparts (Li et al., 2022). PEG-lipids in GO-nanodisks, which preferentially localize to the disk rim, provide steric protection against fusion and self-closure, thereby enhancing structural stability by reducing edge energy (Jurney et al., 2017).

In this study, the mice that were administered LPS presented signs of piloerection, social huddling, and lethargy. Notably, the treatment group displayed significant symptom alleviation. The LPS-treated group displayed pronounced pathological alterations indicative of ALI/ARDS, including thickening of the alveolar walls, increased permeability of the alveolar capillaries, the accumulation of leukocytes within the lung tissue, pulmonary congestion, and endothelial damage. Treatment with GO resulted in a marked improvement in the degree of lung injury induced by LPS. The ratio of the lung tissue weight to volume serves as a metric for assessing the severity of pulmonary oedema. The administration of GO-nanodisks led to a reduction in both the lung coefficient and the W/D ratio in the mice, indicating that GO-nanodisks, may enhance the pulmonary microcirculation and mitigate LPS-induced pulmonary oedema.

Oxidative stress arises from an imbalance between the production of ROS and antioxidant defences within the body. This condition can trigger the aggregation of inflammatory cells and contribute to the development of various severe diseases, including ALI/ARDS (Mondrinos et al., 2014). Patients suffering from ALI/ARDS experience an oxidative burden that results in molecular and cellular damage, primarily due to elevated levels of ROS and inadequate antioxidant protection. In individuals with ALI/ARDS, a notable overproduction of free radicals is observed (Chen et al., 2010). We assessed various antioxidants and products of lipid peroxidation involved in the oxidative stress response to investigate the potential protective effects of GO on LPS-induced lung injury. MDA is a byproduct formed during the oxidation of membrane lipids, which occurs when both plants and animals release significant quantities of superoxide free radicals during oxidative stress. The accumulation of MDA can lead to the cross-linking and aggregation of critical macromolecules, such as proteins and nucleic acids, resulting in cytotoxic effects (Li et al., 2021). SOD is an essential antioxidant enzyme that catalyses the conversion of superoxide anions into hydrogen peroxide (H_2_O_2_) and oxygen (O_2_), thereby mitigating the cellular damage caused by ROS(Halliwell, 2022). CAT, which is widely distributed in biological systems, serves as a primary enzyme for the detoxification of hydrogen peroxide, preventing the formation of highly reactive hydroxyl radicals and protecting cells from oxidative damage. Both SOD and CAT, along with MDA, are integral to the maintenance of cellular redox homeostasis. Moreover, oxidative damage can be mediated not only by ROS derived from molecular oxygen but also through interactions with other reactive species, such as NO. During oxidative stress, cyclooxygenase facilitates the formation of peroxynitrite (ONOO-), a highly reactive compound that can exacerbate cellular damage and oxidative stress (Radi, 2018). In our study, compared with GO alone, GO-nanodisks had a significantly greater antioxidant capacity, as evidenced by an increase in the total antioxidant capacity in mice, elevated activities of SOD and CAT, and reduced levels of MDA and NO.

Organisms have developed a range of defensive strategies to address the challenges posed by ROS, with nuclear factor erythroid 2-related factor 2 (Nrf2) being a prominent mechanism. In pulmonary tissue, Nrf2 is predominantly expressed in epithelial cells and is implicated in various pulmonary disorders, including ARDS, pulmonary fibrosis, asthma, and COPD. Nrf2 is currently recognized as a principal regulator of cellular homeostasis and is essential for maintaining the equilibrium between oxidative stress and antioxidant processes (Boutten et al., 2011; Liang et al., 2019). During the onset of ALI, alveolar macrophages become activated, leading to the binding of Nrf2 to its cis-acting antioxidant response element (ARE). This interaction promotes the expression of downstream phase II detoxification genes, resulting in the production of quinone oxidoreductase 1 (NAD(P)H:quinone oxidoreductase 1, NQO1) and haem oxygenase 1 (HO-1), which serve to mitigate oxidative stress-induced damage (Loboda et al., 2016). *In vivo* studies revealed an increase in the protein expression of HO-1 and NQO1 in the model group. LPS has been shown to stimulate the oxidative stress pathway, thereby activating the Nrf2 pathway. Notably, compared with GO alone, GO-nanodisks more effectively activated the Keap1‒Nrf2 pathway, enhancing the protective effects and increasing the expression of HO-1 and NQO1. Under physiological conditions, Nrf2 is localized primarily in the cytoplasm. GO-nanodisks has the capacity to stimulate the translocation of Nrf2 into the nucleus, thereby increasing its nuclear accumulation. This translocation facilitates the binding of Nrf2 to the antioxidant response element, influencing the expression of redox enzymes and consequently leading to the rapid activation and induction of downstream antioxidant enzymes. This phenomenon may be attributed to the structural similarity between the phospholipid bilayer of GO-nanodiscs and that of cellular membranes, which promotes their effective cellular uptake. Subsequently, the release of GO within the cytoplasm enables its interaction with the Keap1-Nrf2 complex (Ning et al., 2023; Kang et al., 2025).

Chronic oxidative stress has been shown to contribute to the promotion of inflammation. The pathophysiological manifestations of this condition include extensive inflammatory exudation and a disruption in the balance between proinflammatory and anti-inflammatory mediators, which may ultimately culminate in respiratory failure (Li et al., 2020). In the context of lung injury, LPS can initiate the infiltration of inflammatory cells and the subsequent production of inflammatory mediators, including prominent proinflammatory cytokines such as TNF-α, IL-1β, IL-6, and later HMGB-1. The synthesis of these inflammatory mediators facilitates the migration of intravascular neutrophils into lung tissue, thereby exacerbating ALI (Yang et al., 2019; Kim et al., 2014). Consequently, the regulation of inflammatory mediators and chemokines to mitigate the “cascade effect” of inflammatory responses has become a focal point of research aimed at alleviating ALI. Recent studies have indicated that GO can significantly diminish the production of the proinflammatory cytokines TNF-α, IL-1β, and IL-6 in LPS-induced macrophages or murine models, thereby reducing airway inflammation (Marimuthu et al., 2022; Wei et al., 2021). In our investigation, we assessed the levels of the inflammatory factors TNF-α, IL-6, IL-4, and IL-10. Notably, our findings revealed that compared with treatment with LPS, treatment with GO-nanodisks resulted in more pronounced decreases in the levels of proinflammatory factors in the lung tissue and BALF of the mice. Additionally, this treatment was associated with an increase in the secretion of anti-inflammatory factors, the suppression of proinflammatory signaling pathway activation, and a significant reduction in lung inflammation in LPS-induced ALI model mice.

Conversely, NF-κB serves as an important nuclear transcription factor comprising subunits such as p50, p52, and p65. Typically, NF-κB interacts with the inhibitory protein IκBα to form a heterodimer within the cytoplasm. The current consensus indicates that the NF-κB signalling pathway plays a pivotal role in the amplification and regulation of pulmonary inflammation and injury (Dai et al., 2023). For example, the aberrant activation of the NF-κB signalling pathway facilitates the translocation of NF-κB from the cytoplasm to the nucleus, leading to the induction of inflammation-related cytokines, which include proinflammatory factors, chemokines, adhesion molecules, and enzymes that generate secondary inflammatory mediators. This process contributes to the progression of inflammatory diseases and intensifies the inflammatory response (Hussman, 2020). Consequently, the inhibition of the NF-κB pathway activated by LPS may mitigate severe lung tissue damage. Western blot analyses demonstrated that LPS stimulation resulted in the upregulation of p65 and p-p65 protein levels in mice with ALI. However, the application of GO-nanodisks diminished the conversion to the p-p65 protein by obstructing the nuclear translocation of p65 in ALI mice. This intervention helped prevent the persistent activation of the immune response driven by elevated levels of proinflammatory cytokines, such as TNF-α and IL-6, which would otherwise reactivate NF-κB and perpetuate an inflammatory feedback loop that exacerbates the initial inflammatory response (Schnappauf and Aksentijevich, 2020; Wen et al., 2024).

The results of this experiment indicate that, in comparison to free GO, GO-nanodisks demonstrated significant enhancements across various indicators associated with pulmonary edema, oxidative stress, inflammation, and related pathological conditions. These improvements are likely attributable to the biomimetic characteristics of the nanodisk-based carrier system. Typically, the particle size of nanodisks is engineered to range between 100 and 200 nm, aligning with the optimal size parameters for targeted pulmonary delivery (Cooley et al., 2018). Moreover, the delivery efficiency of nanodisks is comparable to, or surpasses, that of conventional liposomal formulations (Ratan et al., 2023). The sustained-release characteristics of nanodisks, exemplified by zero-order release kinetics, facilitate prolonged drug retention within the pulmonary system and help maintain therapeutically effective plasma concentrations (Song et al., 2022). Additionally, nanodisks exhibit enhanced safety profiles; their intrinsic phospholipid matrix confers excellent biocompatibility, thereby mitigating the irritation commonly associated with free GO administration.

## 5 Conclusion

**FIGURE 7:**
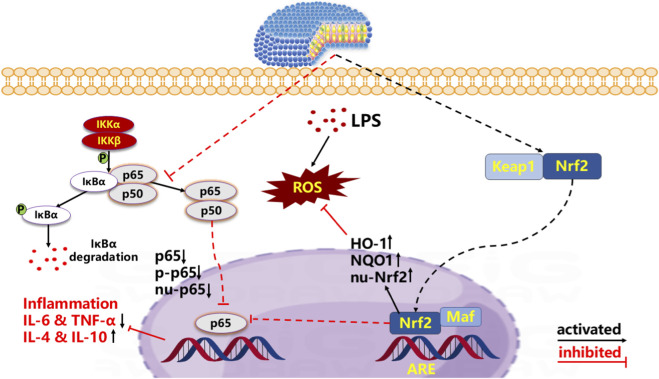
Protective mechanisms of GO-Nanodisks. GO-nanodisks could activate Nrf2-regulated antioxidative pathways and inhibit NF-κB dependent inflammatory response to exert the protective effect against LPS-induced ALI.

In conclusion, we developed a novel formulation of GO nanomedicine and assessed its efficacy through *in vivo* experiments involving LPS-induced ALI in murine models. As shown in Figure 7, our findings indicate that GO-nanodisks significantly enhance the therapeutic effects of GO, contributing to the mitigation of LPS-induced ALI in mice via antioxidant and anti-inflammatory mechanisms. This enhancement may be mediated by the NF-κB and Keap1–Nrf2 signaling pathways. Consequently, our research provides additional evidence and methodologies for investigating the protective effects of natural products against ALI. While certain limitations persist, the results obtained provide a foundational theoretical framework for the future development of GO monomer compound nanomedicines and further investigations into their protective effects against lung injury.

## Data Availability

The original contributions presented in the study are included in the article/Supplementary Material, further inquiries can be directed to the corresponding author.

## Abbreviations

ALI, acute lung injury; ARDS, acute respiratory distress syndrome; MODS, multiple organ dysfunction syndrome; SIRS, systemic inflammatory response syndrome; ICUs, intensive care units; COPD, chronic obstructive pulmonary disease; GO, garlic oil; TEM, transmission electron microscopy; HPLC, high-performance liquid chromatography; BALF, bronchoalveolar lavage fluid; H&E, haematoxylin and eosin; TNF-α, tumour necrosis factor-alpha; IL-4, interleukin-4; IL-6, interleukin-6; IL-10, interleukin-10; T-AOC, total antioxidant capacity; SOD, superoxide dismutase; MDA, malondialdehyde; H2O2, hydrogen peroxide; SDS–PAGE, sulfate‒polyacrylamide gel electrophoresis; PVDF, polyvinylidene fluoride; ECL, enhanced chemiluminescence; Nrf2, nuclear factor erythroid 2-related factor 2; ARE, antioxidant response element.
