# Supplementary figures and images for: Garlic oil-loaded nanodisks for the amelioration of acute lung injury via modulation of the NF-κB and Keap1–Nrf2 axis
Source: Front Mol Biosci. 2026 Jan 5;12:1686436. doi: 10.3389/fmolb.2025.1686436 (PMC12812721; doi:10.3389/fmolb.2025.1686436)

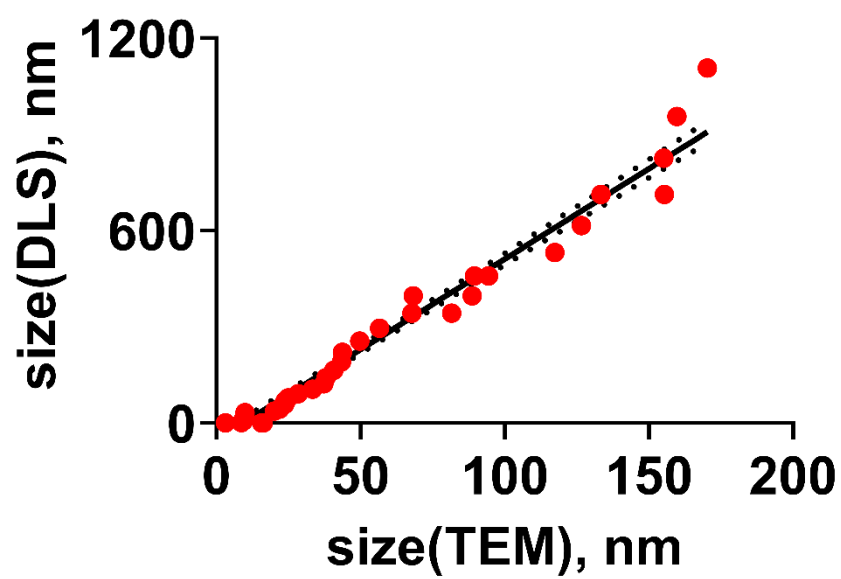

FIGURE. S1 The correlation between particle sizes determined using DLS and TEM for GO-nanodisks

Supplement: Supplementary file 1 [file DataSheet1.pdf]

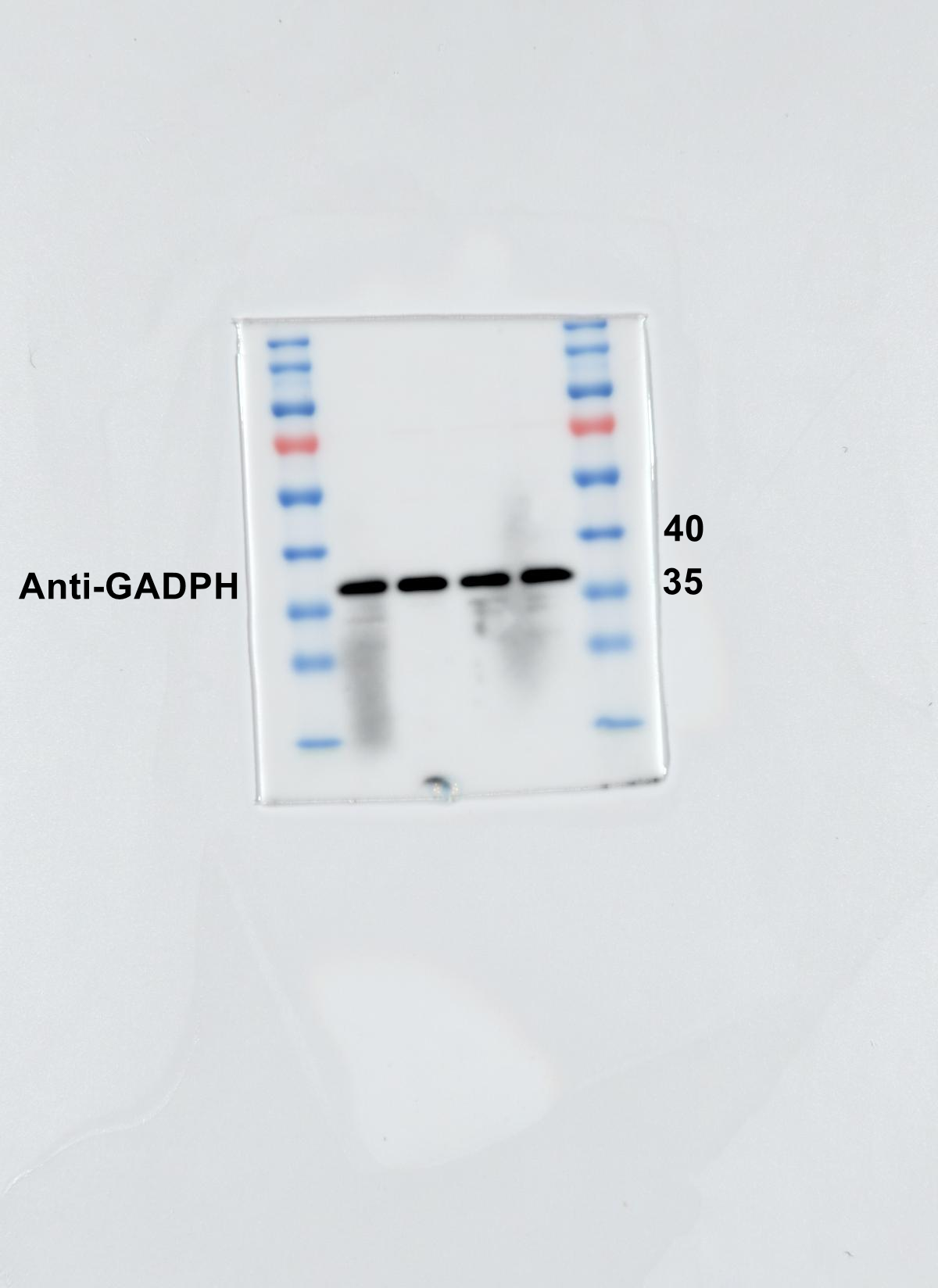

Supplement: Supplementary file 2 [file DataSheet2.zip › Image 4.TIF]

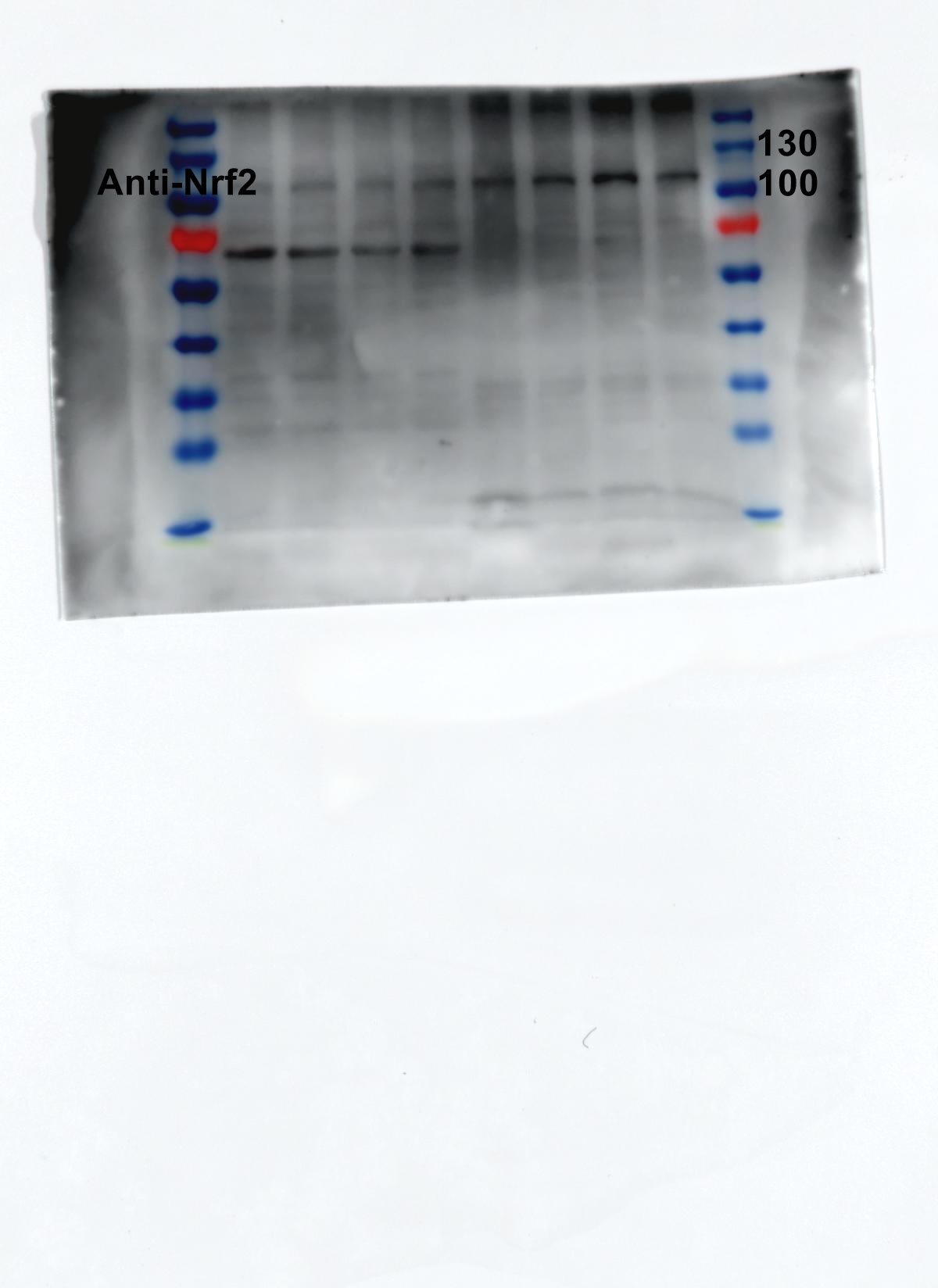

Supplement: Supplementary file 2 [file DataSheet2.zip › Image 5.TIF]

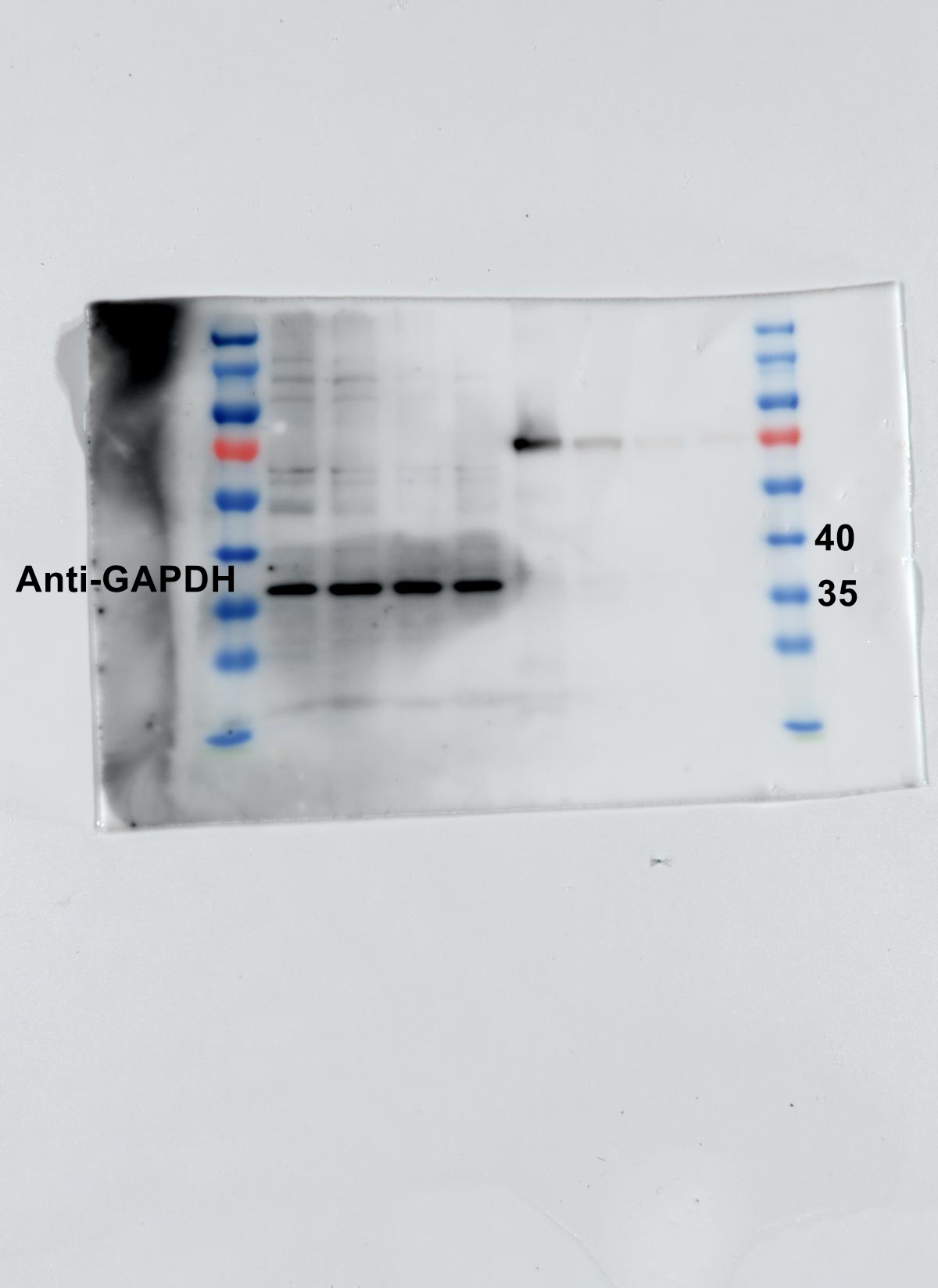

Supplement: Supplementary file 2 [file DataSheet2.zip › Image 6.TIF]

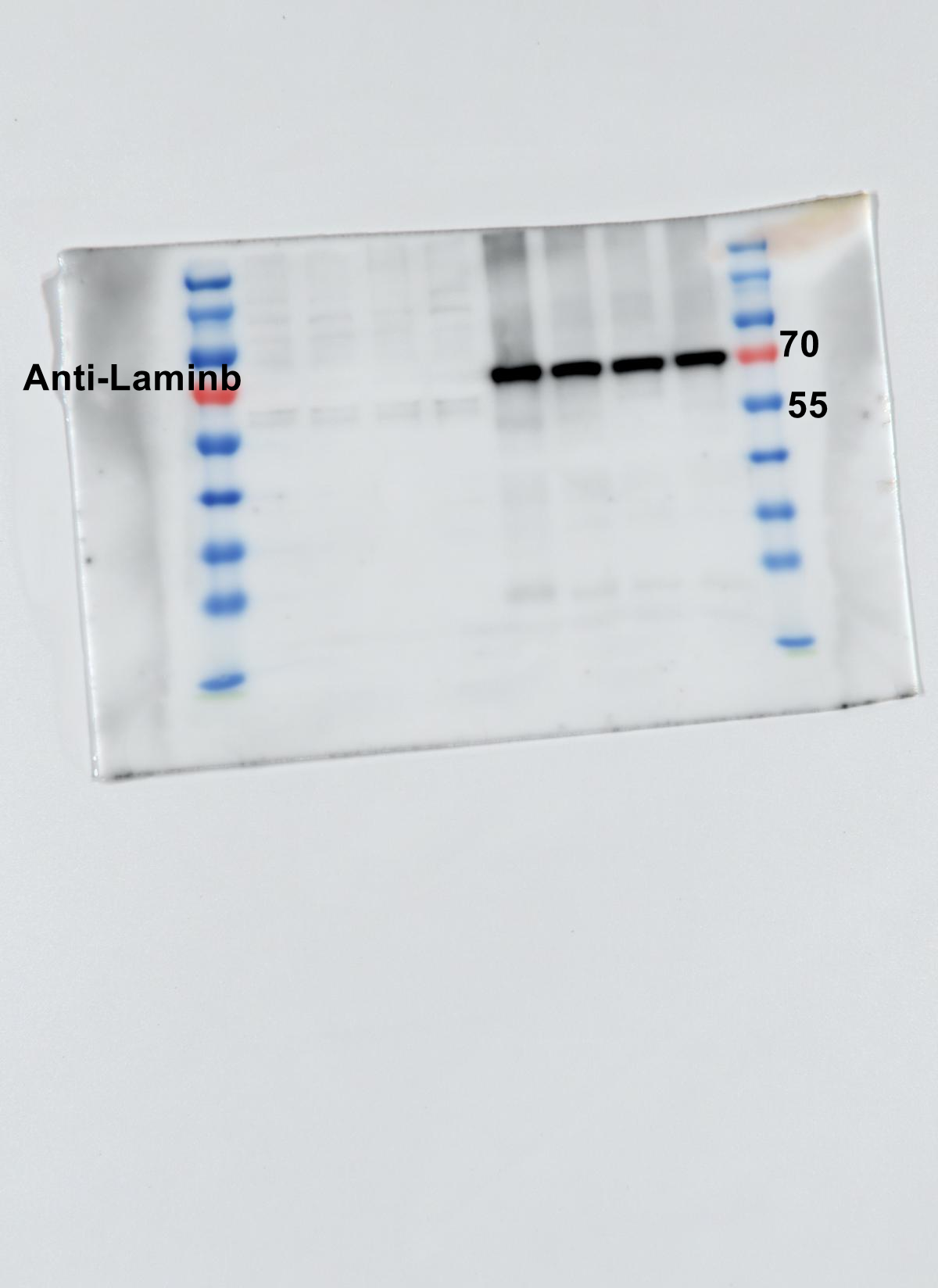

Supplement: Supplementary file 2 [file DataSheet2.zip › Image 7.TIF]

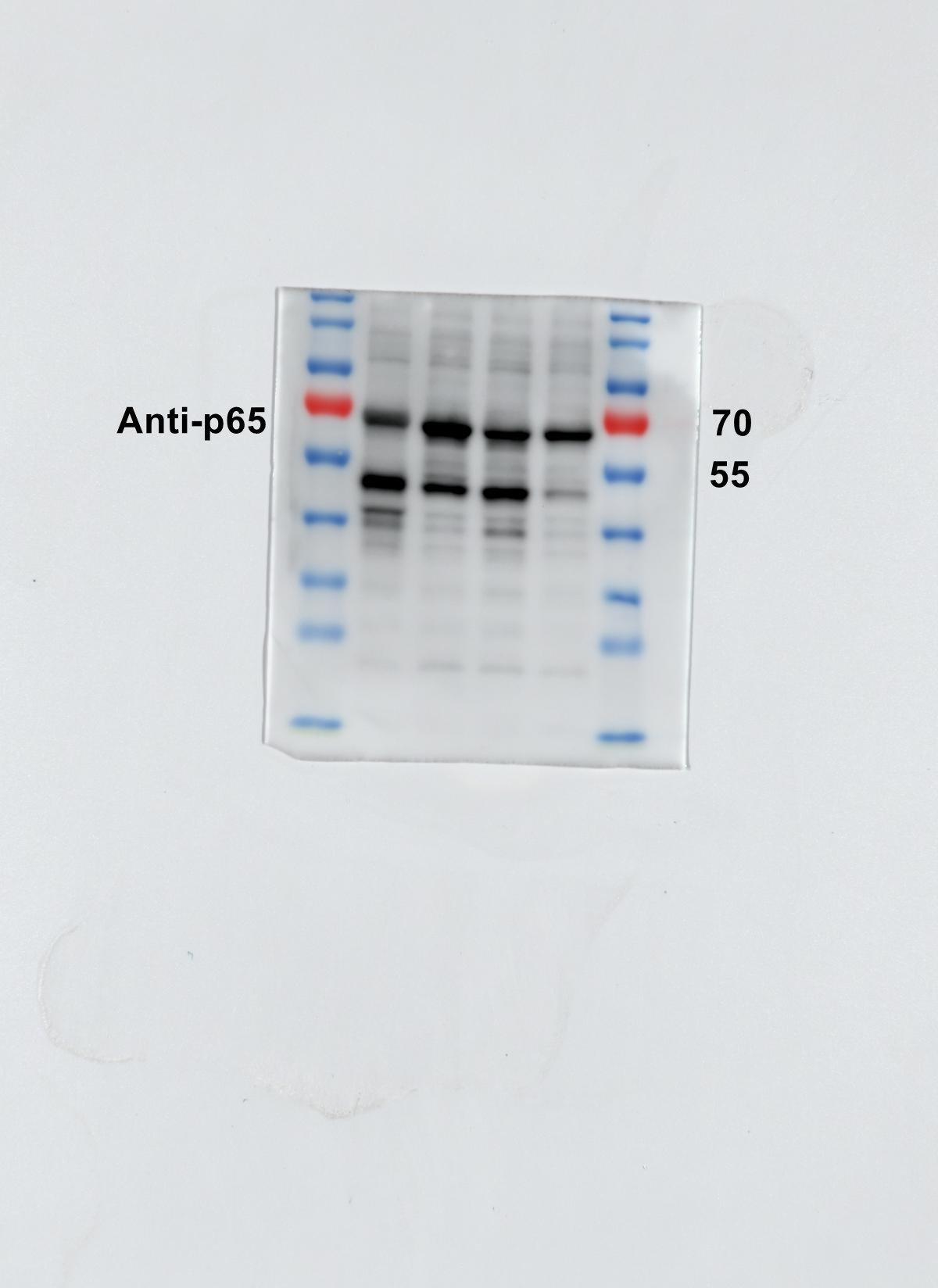

Supplement: Supplementary file 2 [file DataSheet2.zip › Image 8.TIF]

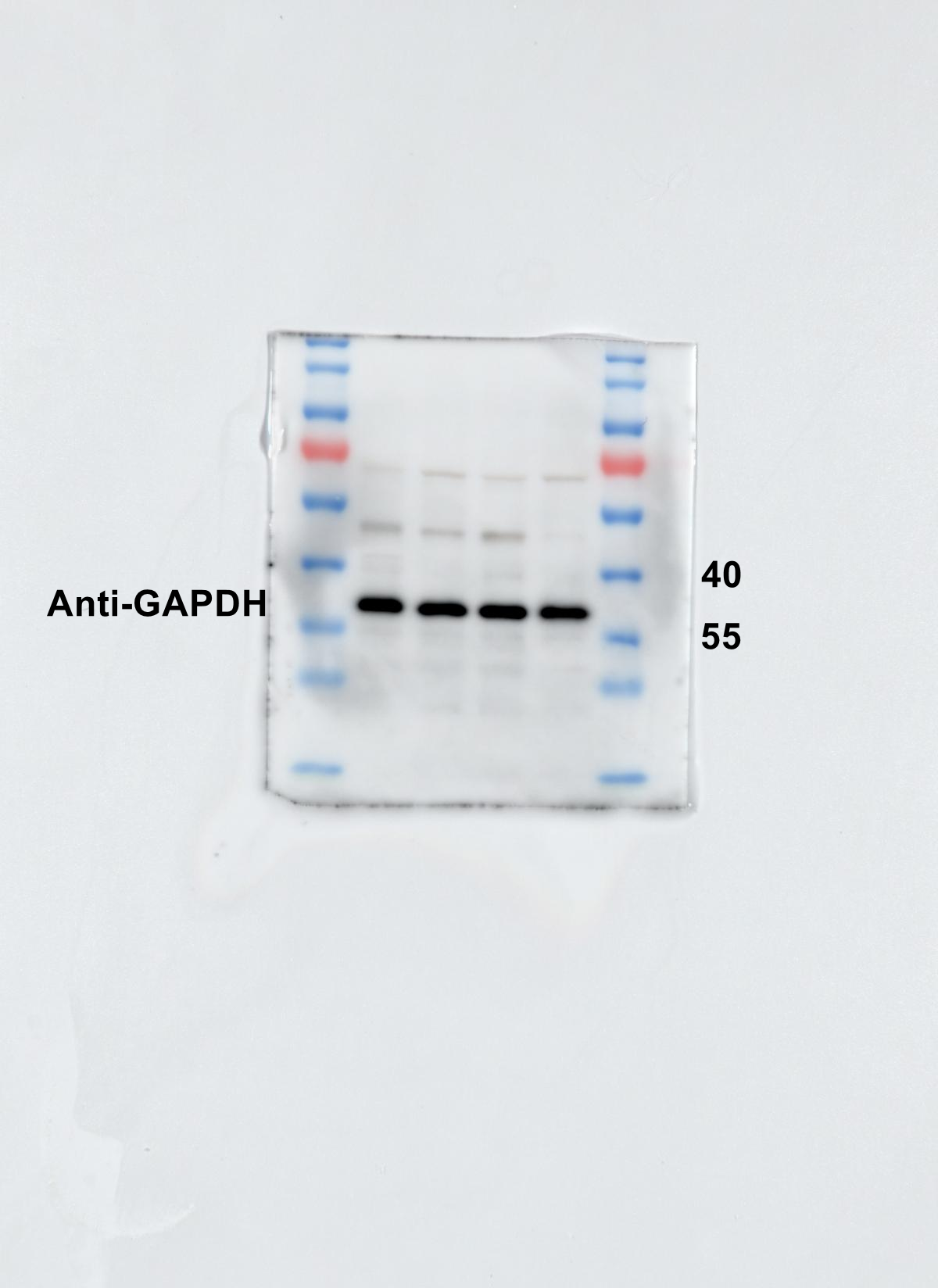

Supplement: Supplementary file 2 [file DataSheet2.zip › Image 9.TIF]

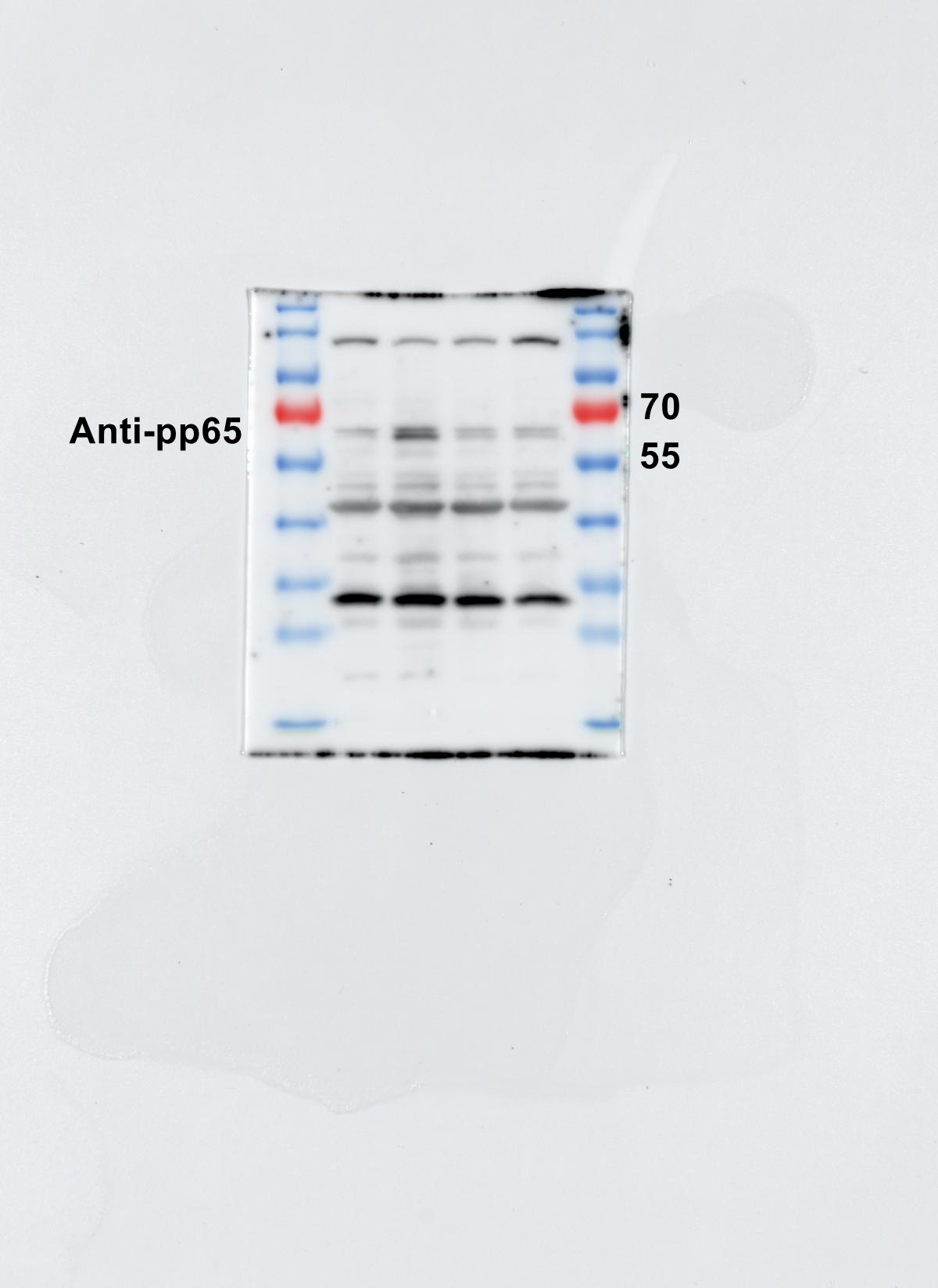

Supplement: Supplementary file 2 [file DataSheet2.zip › Image 10.TIF]

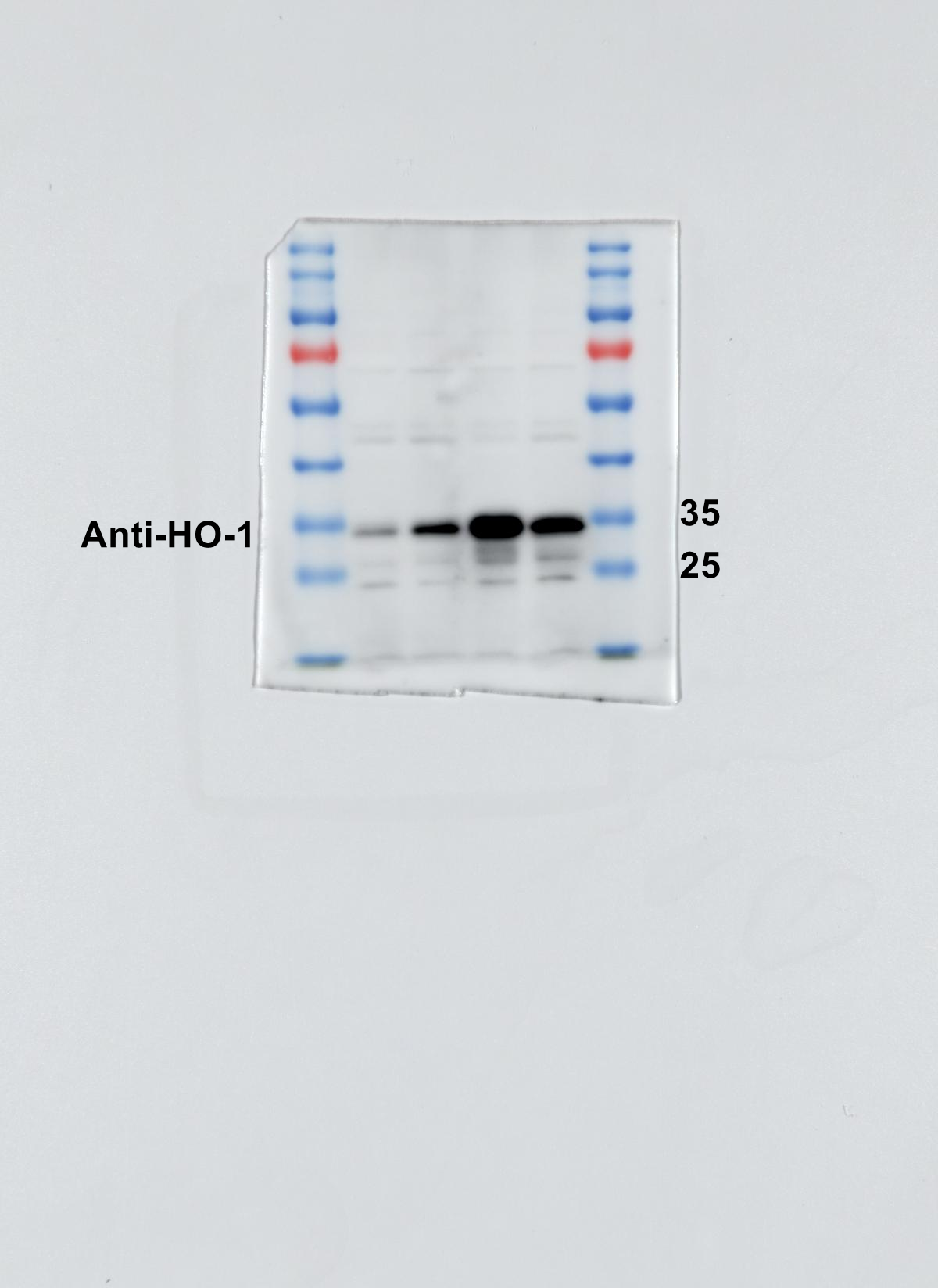

Supplement: Supplementary file 2 [file DataSheet2.zip › Image 1.TIF]

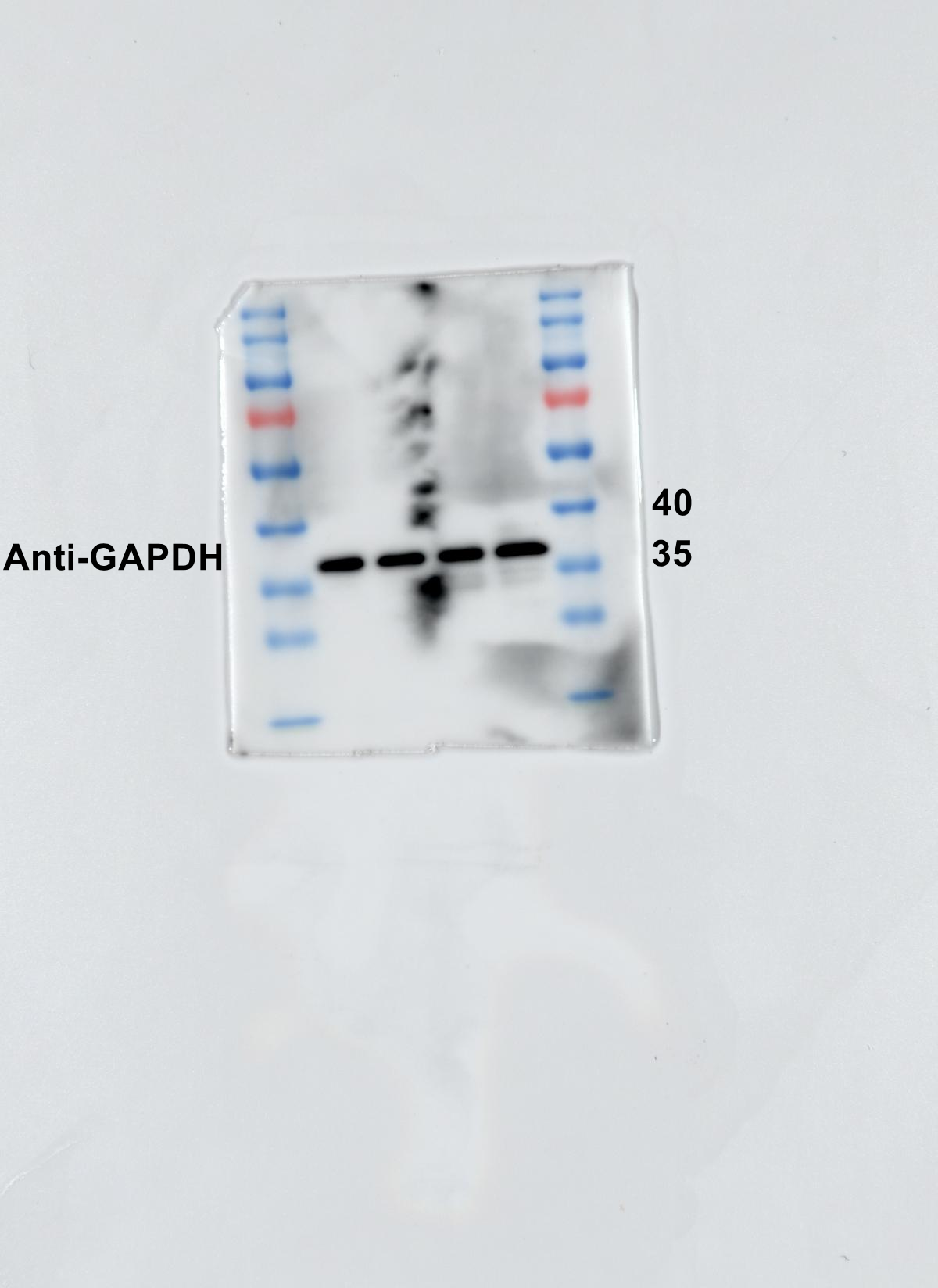

Supplement: Supplementary file 2 [file DataSheet2.zip › Image 2.TIF]

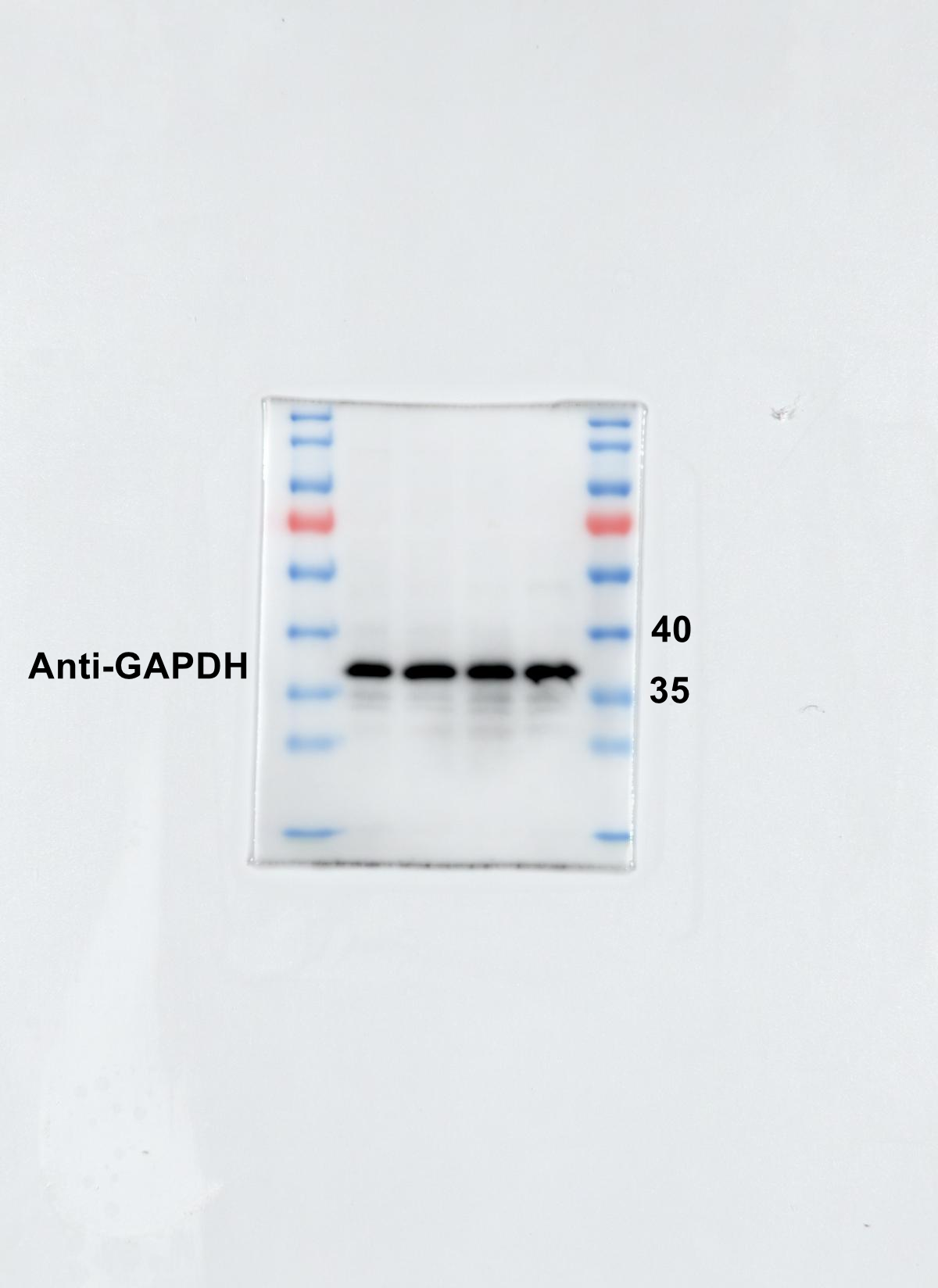

Supplement: Supplementary file 2 [file DataSheet2.zip › Image 11.TIF]

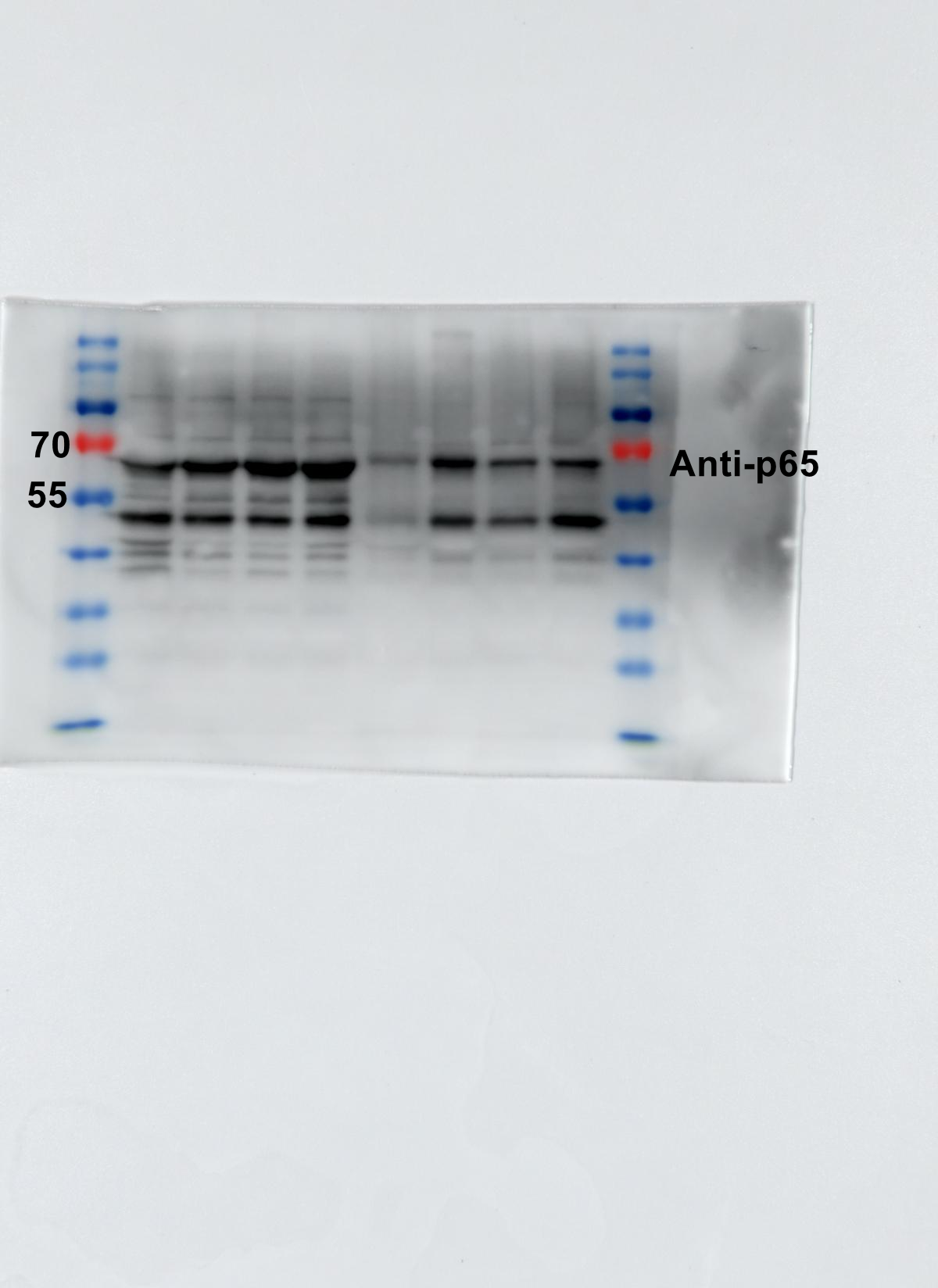

Supplement: Supplementary file 2 [file DataSheet2.zip › Image 12.TIF]

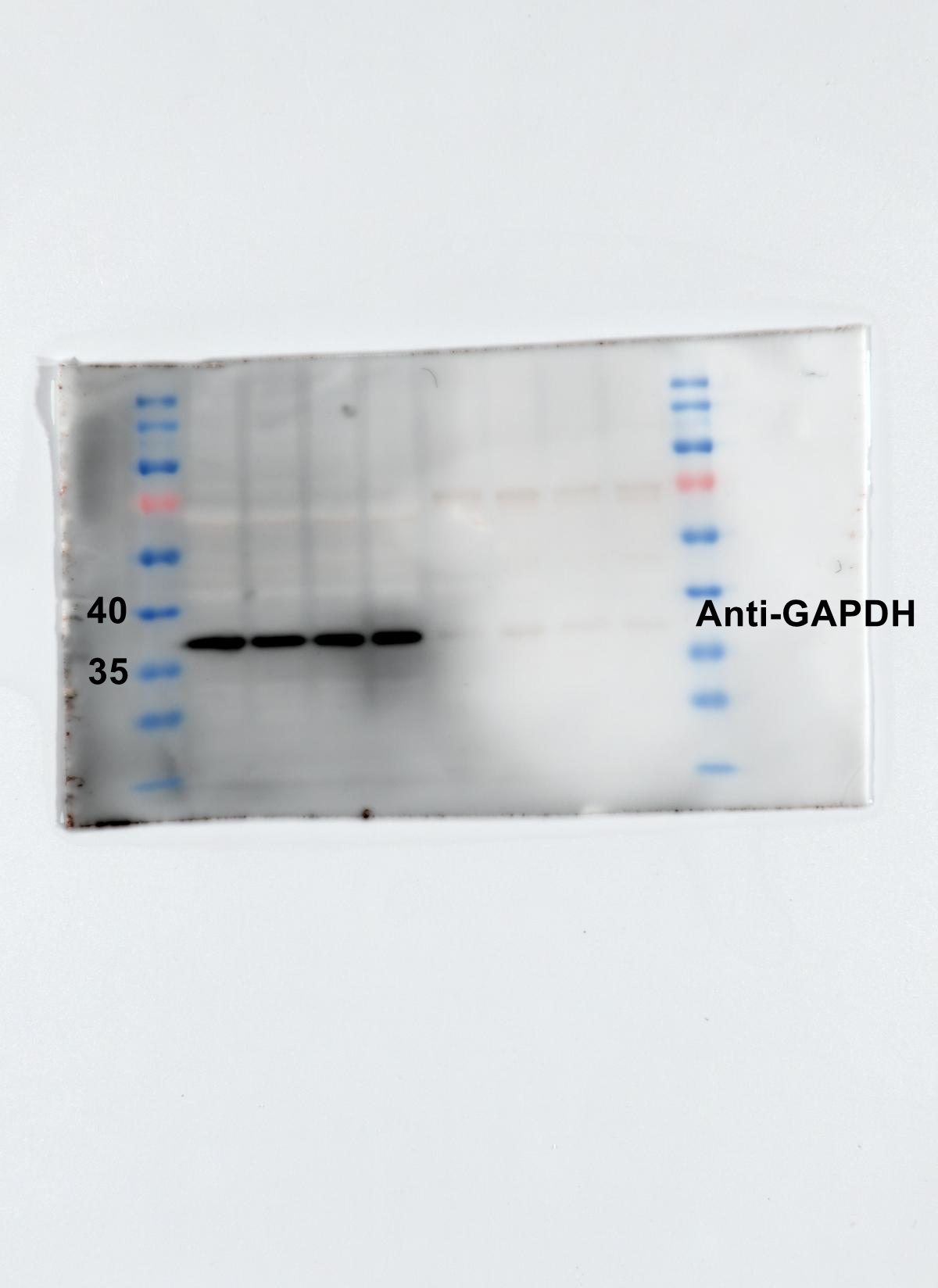

Supplement: Supplementary file 2 [file DataSheet2.zip › Image 13.TIF]

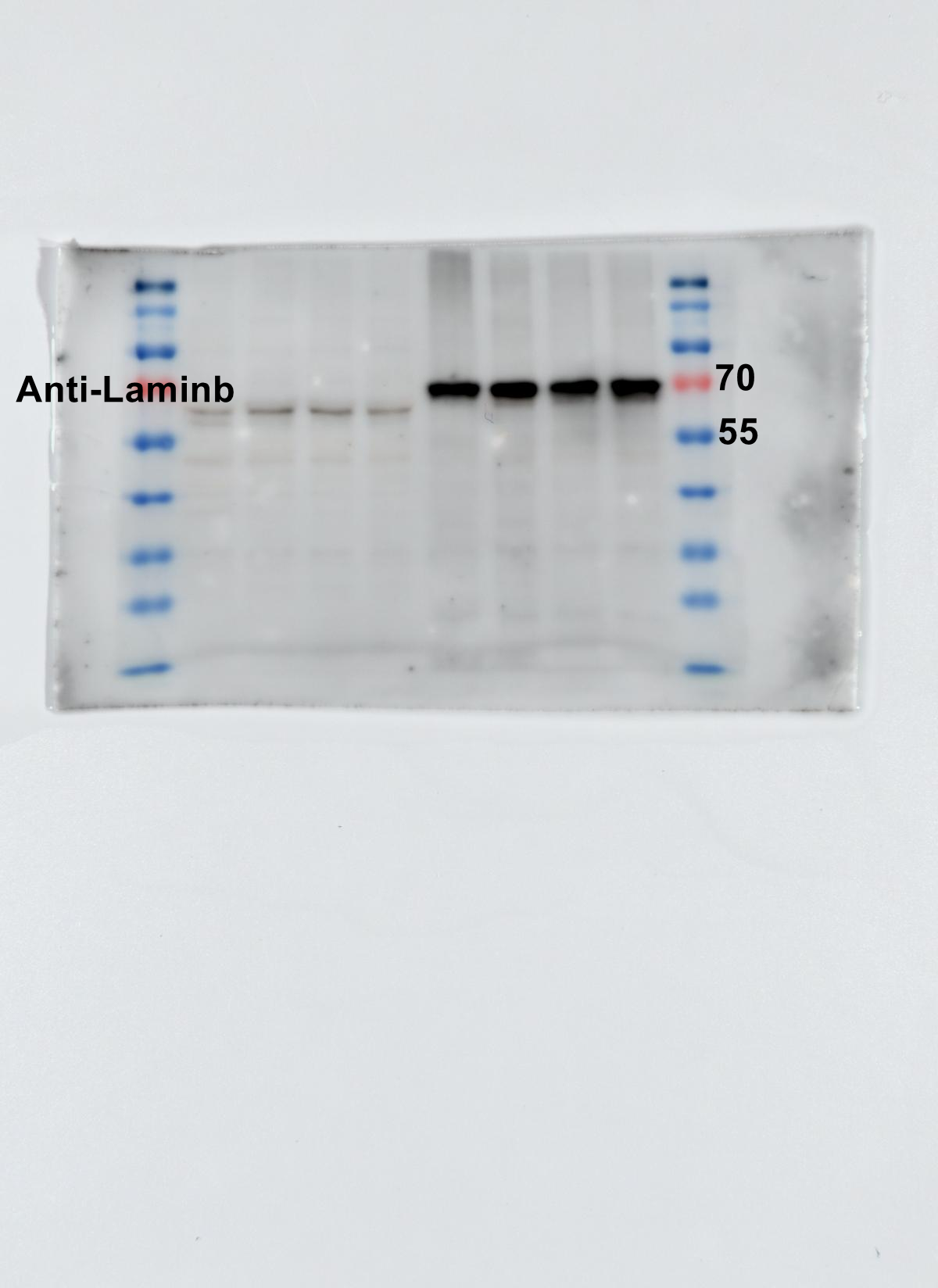

Supplement: Supplementary file 2 [file DataSheet2.zip › Image 14.TIF]

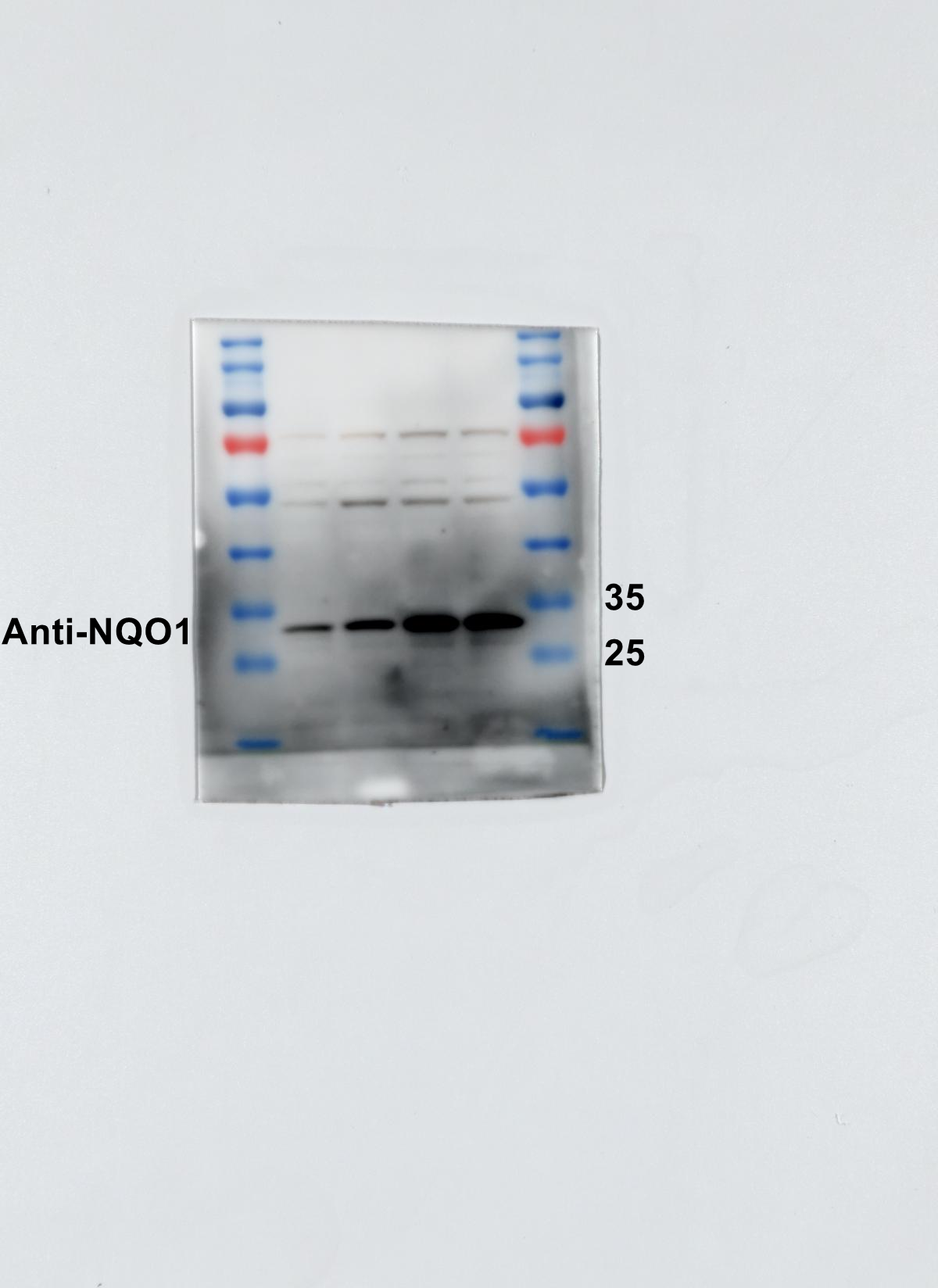

Supplement: Supplementary file 2 [file DataSheet2.zip › Image 3.TIF]
